# Integrative Analysis of Transgenic Alfalfa (*Medicago sativa* L.) Suggests New Metabolic Control Mechanisms for Monolignol Biosynthesis

**DOI:** 10.1371/journal.pcbi.1002047

**Published:** 2011-05-19

**Authors:** Yun Lee, Fang Chen, Lina Gallego-Giraldo, Richard A. Dixon, Eberhard O. Voit

**Affiliations:** 1 Integrative BioSystems Institute and The Wallace H. Coulter Department of Biomedical Engineering, Georgia Institute of Technology and Emory University, Atlanta, Georgia, United States of America; 2 BioEnergy Sciences Center (BESC), Oak Ridge, Tennessee, United States of America; 3 Plant Biology Division, Samuel Roberts Noble Foundation, Ardmore, Oklahoma, United States of America; Stanford University, United States of America

## Abstract

The entanglement of lignin polymers with cellulose and hemicellulose in plant cell walls is a major biological barrier to the economically viable production of biofuels from woody biomass. Recent efforts of reducing this recalcitrance with transgenic techniques have been showing promise for ameliorating or even obviating the need for costly pretreatments that are otherwise required to remove lignin from cellulose and hemicelluloses. At the same time, genetic manipulations of lignin biosynthetic enzymes have sometimes yielded unforeseen consequences on lignin composition, thus raising the question of whether the current understanding of the pathway is indeed correct. To address this question systemically, we developed and applied a novel modeling approach that, instead of analyzing the pathway within a single target context, permits a comprehensive, simultaneous investigation of different datasets in wild type and transgenic plants. Specifically, the proposed approach combines static flux-based analysis with a Monte Carlo simulation in which very many randomly chosen sets of parameter values are evaluated against kinetic models of lignin biosynthesis in different stem internodes of wild type and lignin-modified alfalfa plants. In addition to four new postulates that address the reversibility of some key reactions, the modeling effort led to two novel postulates regarding the control of the lignin biosynthetic pathway. The first posits functionally independent pathways toward the synthesis of different lignin monomers, while the second postulate proposes a novel feedforward regulatory mechanism. Subsequent laboratory experiments have identified the signaling molecule salicylic acid as a potential mediator of the postulated control mechanism. Overall, the results demonstrate that mathematical modeling can be a valuable complement to conventional transgenic approaches and that it can provide biological insights that are otherwise difficult to obtain.

## Author Summary

Cellulose-based biofuels presently offer the most environmentally attractive and technologically promising alternative to fossil fuels. To be viable, biofuels must be derived from non-food crops, such as grasses, wood, bark, and plant residues. Techniques for releasing the energy stored in these renewable materials must first untangle a very recalcitrant scaffold of interlinking molecules inside the plant cell walls, which is very costly. Much of the recalcitrance is due to the natural polymer lignin, which hardens the cell walls and is composed of three different building blocks, called monolignols. Modern transgenic techniques have yielded plant lines whose cell walls are easier to break down, but some of these modified plants have exhibited unexplained and undesired features. Here, we present new computational methods for analyzing monolignol biosynthesis in unprecedented detail. The analysis simultaneously accounts for lignin biosynthesis in various transgenic lines and different developmental stages and yields six novel, testable postulates regarding the metabolic control of the pathway. The results suggest new, targeted experiments towards a better understanding of monolignol biosynthesis and issues of recalcitrance reduction. More generally, the results highlight the genuine benefits of using computational methods as companions and complements to experimental studies.

## Introduction

The complex, interwoven structure of lignin, cellulose, and hemicellulose polymers in plant cell walls is the main cause for the recalcitrance of lignocellulosic feedstocks to microbial and enzymatic deconstruction towards fermentable sugars. This recalcitrance, in turn, accounts for the high cost of biofuel production from renewable sources [1]. In current technologies, the release of polysaccharides from the entanglement with lignin demands a thermo-chemical pretreatment that is expensive and has undesirable side effects during the later fermentation steps. Recent efforts aimed at decreasing the lignin content with transgenic techniques suggest that it might be feasible to reduce or even obviate the need for pretreatment [2], which would permit the inclusion of polymer separation in downstream biomass processing technologies [3] and thereby make the cost of biofuel production competitive with that of fossil fuels.

Reflecting the substantial impact of lignin on forage digestibility [4], pulping efficiency [5] and sugar release from biomass [2], considerable effort has been devoted towards a better understanding of monolignol biosynthesis *in situ*. Most pertinent genes have been identified in model species with complete sequence information, and knowledge from these genomes is currently being used for homology searches in species where sequencing efforts are ongoing. Such homology investigations are often effective, but caution is necessary, because multiple genes with similar sequences and annotations pointing to the same enzyme may possess distinct expression patterns and substrate preferences [5]. As a consequence, monolignol biosynthesis *in vivo* might be strikingly different among species and depend not only on gene sequences, but also on the tissue or even cell type of interest. Thus, before genetic modification strategies that had proven effective in some species are implemented in another species, it is prudent to consider and account for contextual differences. As a case in point, alfalfa (*Medicago sativa* L.), the organism used for our analysis, exhibits substantial differences in cell wall composition among the different internodes of young plants.

**Figure 1:**
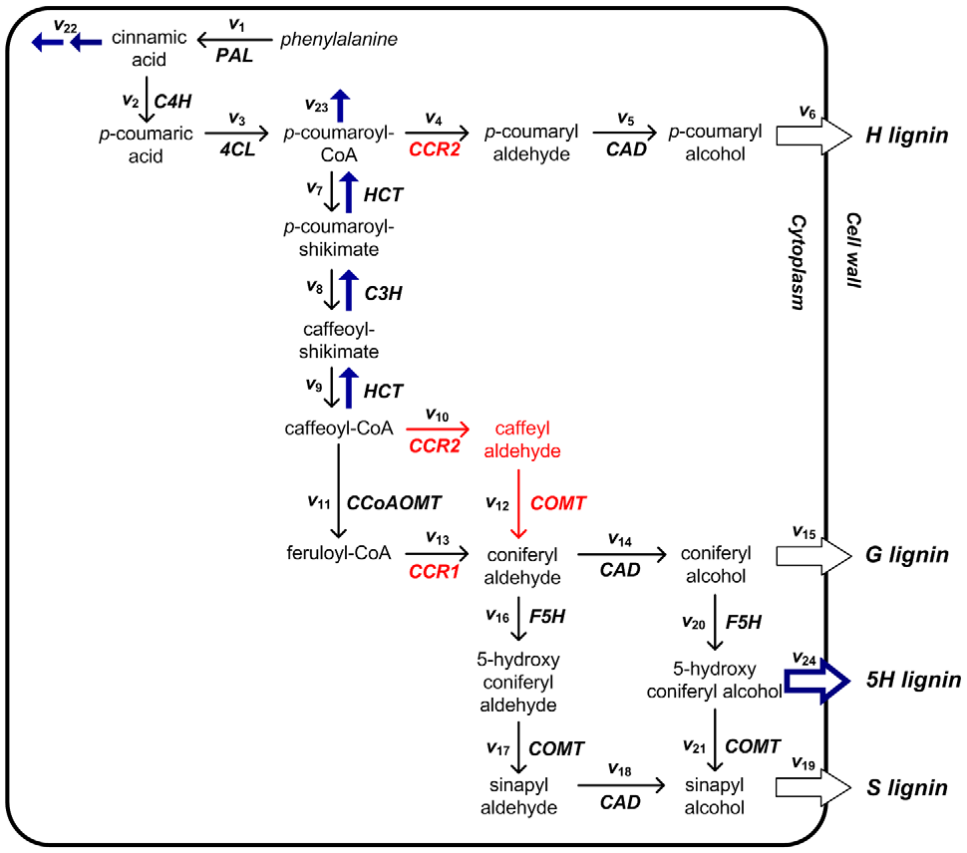
Successive amendments of the metabolic pathways and transport processes leading to four hydroxycinnamyl alcohols. The commonly accepted pathway of monolignol biosynthesis, which produces *p*-hydroxyphenyl (H), guaiacyl (G), 5-hydroxyconiferyl (5H), and syringyl (S) lignin monomers, is presented in black, with solid arrows representing metabolic conversions and open arrows collectively representing all events during the transport of monolignol precursors into the cell wall. Important revisions suggested by the recent identification of two CCR isoforms—CCR1 and CCR2—are colored in red and discussed in the text. Arrows colored in blue represent additional reactions and transport processes that are probably negligible in wild-type plants but found to become significant in some transgenic strains. Abbreviations for enzymes: PAL, phenylalanine ammonia-lyase; C4H, cinnamate 4-hydroxylase; 4CL, 4-coumarate:CoA ligase; CCR, cinnamoyl-CoA reductase; CAD, cinnamyl alcohol dehydrogenase; HCT, hydroxycinnamoyl CoA:shikimate hydroxycinnamoyl transferase; C3H, *p*-coumarate 3-hydroxylase; CCoAOMT, caffeoyl-CoA *O*-methyltransferase; COMT, caffeic acid *O-*methyltransferase; F5H, ferulate 5-hydroxylase.

In most woody plants, the biochemical pathway of monolignol biosynthesis leads to three building blocks of lignin, which are known as *p*-hydroxyphenyl (H), guaiacyl (G) and syringyl (S) monolignols (Figure 1). In potential bioenergy crops like poplar and switchgrass, lignin consists principally of G and S units, while H units are present in low to negligible quantities. In other plants, including some alfalfa transgenics, H can be present in significant amounts. Although the generic sequences of metabolic reactions within the monolignol pathway have been identified, it is becoming increasingly clear that critical details of the pathway structure and its regulation are not entirely understood. As a case in point, Chen *et al.* [6] recently introduced systematic, transgenic alterations in alfalfa (*Medicago sativa* L.) plants by independently modifying the activities of seven key enzymes of monolignol biosynthesis. While many of the results were easily explained, down-regulation of caffeoyl coenzyme A 3-*O*-methyltransferase (CCoAOMT) had little effect on S lignin, an observation that is conceptually inconsistent with the commonly accepted pathway structure (Figure 1; black colored arrows). A recent study identified two isoforms of cinnamoyl CoA reductase (CCR), MtCCR1 and MtCCR2, in *Medicago truncatula* [7]. Furthermore, an earlier finding had suggested that caffeyl aldehyde is one of the preferred substrates for caffeic acid 3-*O*-methyltransferase (COMT) in alfalfa [8]. Taken together, these findings could imply an alternative route for S lignin synthesis (Figure 1; red colored arrows) upon CCoAOMT down-regulation [8], [9]. However, they cannot explain why only G lignin is decreased because feruloyl-CoA is a common precursor of both G and S lignin.

In dicotyledonous plants like alfalfa, the stem consists of many segments, called *internodes*. During maturation, all internodes grow asynchronously and thus independently represent different developmental stages. This phenomenon suggests a customized modeling approach: Instead of studying the pathway within a single developmental context, it seems advantageous to launch a systematic investigation that simultaneously encompasses dozens of internodes from seven wild-type or transgenic plants. This comprehensive approach supports the fact that lignin biosynthesis is tightly coordinated by a hierarchy of transcription factors during secondary wall thickening [10]. It also circumvents the potential problem that regulatory mechanisms might escape discovery during an analysis based on singular phenotypic datasets, such as lignin content and monomer composition, if only one internode or one transgenic line is studied at a time. This potential failure to detect regulatory signals is exacerbated in the lignin system by the fact that several enzymes in the pathway catalyze multiple steps, which makes intuitive analyses difficult.

With a comprehensive analysis of several datasets as the target, we propose here a novel modeling approach that integrates the data in a semi-dynamic fashion. First, flux balance analysis (FBA) [11] is applied independently in each individual internode of the wild-type plant. In contrast to microbial systems, where maximization of the growth rate is usually assumed to be the species' overall objective, we use the monolignol production as the objective function for FBA. Second, for every internode of a lignin-modified line, we use the method of minimization of metabolic adjustment (MOMA) [12] to characterize the altered flux distribution in relation to the corresponding FBA solution for the same wild-type internode. Specifically, the relative proportions of the fluxes leading to three lignin monomers are constrained at experimentally-observed values to improve the prediction. Finally, we perform a Monte Carlo-like simulation of randomly parameterized kinetic models in cases where the results arising from the static models may have alternative, kinetics-based explanations.

This combined modeling approach represents, to the best of our knowledge, the first computational study of lignin biosynthesis in angiosperm stem tissues and, more generally, of secondary plant metabolism in angiosperms. As we will discuss later, the model analysis resulted in six postulates concerning the metabolic control of monolignol biosynthesis that had not been considered at all or at least not in detail. These postulates address the reversibility of some enzymatic reactions, shed light on the hypothesis of independent pathways for the synthesis of G and S monolignols, and suggest a novel feedforward regulatory mechanism exerted by a cinnamic acid-derived compound. Of note is the fact that evidence in support of this last postulate has subsequently been obtained in laboratory experiments. By critically evaluating the transgenic data against a revised pathway structure in alfalfa, we hope these postulates will not only serve as guidelines for directing future experiments, but also provide mechanistic insights that will aid the design of combined genetic modification strategies toward the generation of bioenergy crops with reduced recalcitrance.

## Results

### FBA-guided elucidation of three principal branch points

Accounting for recent experimental observations, we adopted a revised pathway structure of monolignol biosynthesis in alfalfa stems that includes the CCR2-catalyzed reduction of caffeoyl-CoA to caffeyl aldehyde and the subsequent synthesis of coniferyl aldehyde by COMT (Figure 1: black and red colored reactions), as explained earlier.

**Figure 2:**
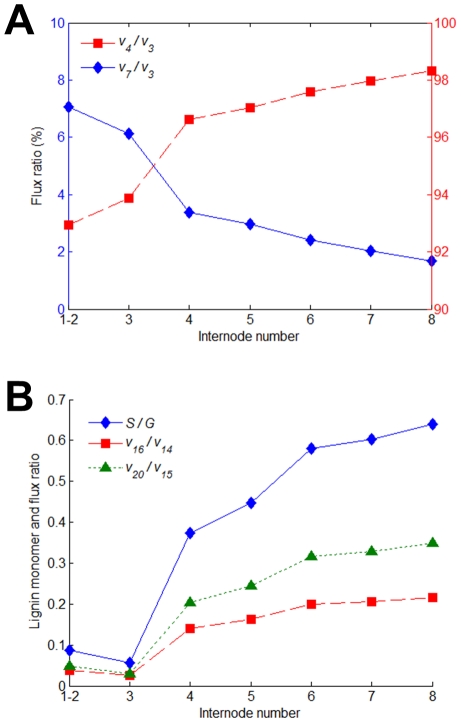
Flux partitioning at principal branch points. (A) Developmental patterns of flux partitioning at *p*-coumaroyl CoA branch point in wild-type plants, given as percentage of *v*_3_. (B) Comparison of flux partitioning at coniferyl aldehyde (*v*_16_/*v*_14_) and coniferyl alcohol (*v*_20_/*v*_15_) branch points with the ratio of S to G lignin (S/G) in individual internodes of wild-type plants.

The pathway of monolignol biosynthesis contains a fairly small number of branch points, and it is known that flux partitioning at these branch points determines the ultimate transport fluxes *v*_6_, *v*_15_ and *v*_19_ and thus the relative amounts of lignin monomers (*cf.* [13]). The FBA-derived steady-state flux analysis for wild-type plants supports this argument. It suggests that variation in lignin composition from young to mature internodes is accomplished by modulating the flux partitioning at three principal branch points: *p*-coumaroyl-CoA, coniferyl aldehyde, and coniferyl alcohol. As a paradigm illustration, the proportion of H lignin declines from 7% of the total monomer yields in the first two internodes to 1% in the eighth internode. This decline is singularly achieved through a monotonic decrease in *v*_4_ (Figure 2A). A parallel increase in the ratio of S to G lignin—commonly termed the S/G ratio—from 0.09 in the first two internodes to 0.64 in the eighth internode requires a combined effort of flux adjustments at coniferyl aldehyde and coniferyl alcohol (Figure 2B). Since F5H controls the first committed steps (*i.e.*, *v*_16_ and *v*_20_) towards the synthesis of S lignin, one would expect to see its expression being up-regulated in mature versus young internodes, which has recently been validated by microarray analysis (Table 4 of [14]).

### Minor extension of the pathway structure

For a systemic analysis of the pathway we used the results of a gene modification study in alfalfa where genes encoding for PAL, C4H, HCT, C3H, CCoAOMT, F5H, and COMT were independently down-regulated. With the exception of F5H-modified lines, which did not permit measurements of the targeted enzyme activity, we applied MOMA to each strain and each internode and predicted the new steady-state flux distribution (see *Materials and Methods*).

A very interesting result is the fact that no feasible solution exists for four of the six transgenic plants, if the revised metabolic map is correct (Figure 1; black and red colored arrows). For example, if C4H activity is down-regulated to 45% of its wild-type level, it is analytically impossible to derive a set of fluxes that satisfies the mass balance at cinnamic acid as well as the observed lignin composition, if the supply of phenylalanine is constant. To remedy this situation, it seems to be necessary to add to the pathway structure three “overflow” fluxes counteracting the potential accumulation of the intermediate metabolites cinnamic acid, *p*-coumaryl aldehyde, and 5-hydroxyconiferyl alcohol (blue arrows *v*_22_, *v*_23_, *v*_24_ in Figure 1). This proposed amendment is at least partially supported by observations. First, salicylic acid (SA), an essential signaling molecule for systemic acquired resistance against pathogen attack, can be formed from cinnamic acid [15], [16], [17], although it may also originate from the shikimate pathway via isochorismate [18]. Second, the biosynthesis of all flavonoids begins with the condensation of *p*-coumaroyl-CoA and three molecules of malonyl-CoA by the enzyme chalcone synthase [19]. And third, incorporation of 5-hydroxyconiferyl alcohol into lignin polymer is found in the COMT-deficient alfalfa [20]. Thus, we included these additional effluxes, and the expanded system (Figure 1; *v*_1_ to *v*_24_) permitted feasible solutions in all cases tested.

In wild-type plants, the FBA-derived steady-state values of the three added fluxes are minimized to prevent lignin precursors from being channeled into peripheral pathways producing SA or flavonoids. In the transgenic plants, these auxiliary fluxes are no longer restricted to small values and thus can be raised to substantial levels to facilitate the re-distribution of fluxes. However, the assumption that the peripheral fluxes are minimized in wild-type plants must be handled with caution: although the phenylpropanoid pathway in cells undergoing secondary wall thickening may evolve towards maximizing the synthesis of lignin precursors, this is apparently not the case when biosynthesis of flavonoid-derived products, which may function as floral pigments or as anti-microbial agents, becomes the plant's top priority.

### Trends in flux patterns

**Figure 3:**
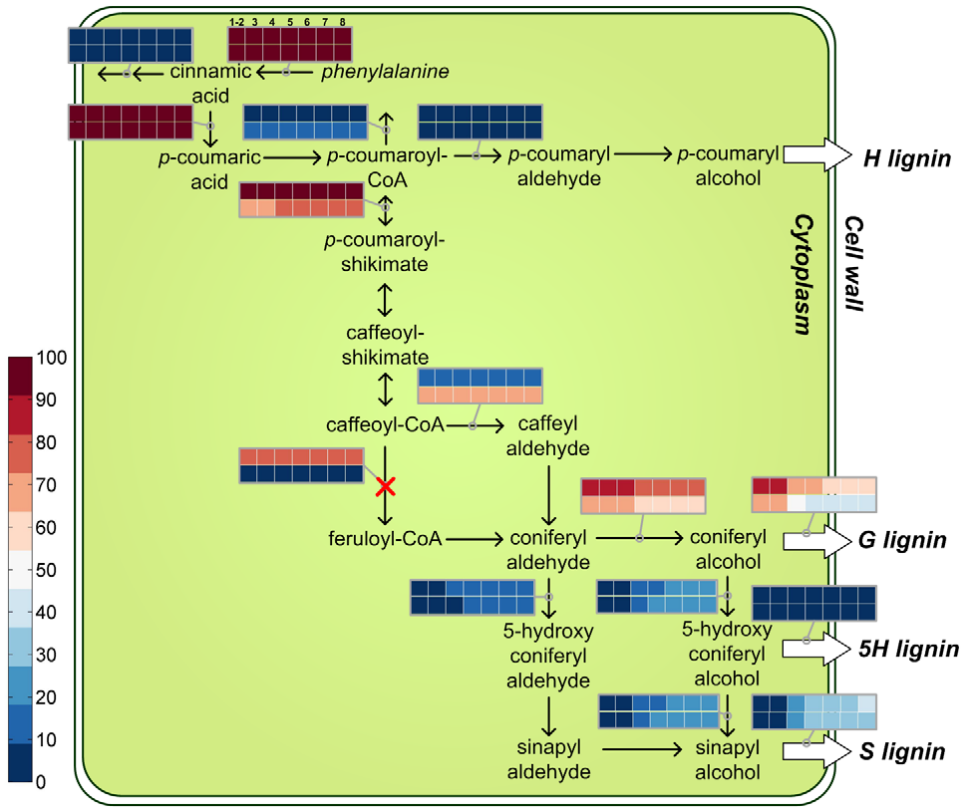
Developmental evolution of the steady-state flux distribution in CCoAOMT-deficient plants versus the wild-type plants. The reaction crossed out in red is dysfunctional in this particular transgenic strain. Two rows of colored boxes are placed either above horizontally plotted fluxes, or to the left of vertically plotted fluxes. The first row represents wild-type plants, whereas the second row refers to transgenic line (here a CCoAOMT-deficient plant). Each row contains seven colored boxes, which represent the seven stem internodes (with internodes 1 and 2 merged). In FBA and MOMA, all fluxes are normalized to the initial step in the pathway, namely the conversion of phenylalanine to cinnamic acid. Therefore, the color of each box shows the normalized steady-state value of the corresponding flux in one specific internode: low values are dark blue, intermediate values are white, and high values are dark red. Because all the reactions along a linear pathway have the same flux values at steady state, only the first one is shown.

The MOMA analysis revealed flux distributions for all transgenic lines and their individual internodes. Figure 3 shows the developmental evolution of flux patterns in CCoAOMT-deficient plants; similar plots for other transgenic plants are given in Figures S1, S2, S3, S4, and S5. Of note is that all computed fluxes exhibit strong and essentially monotonic trends: for each transgenic line, the flux partitioning at important branch points follows clear trends throughout the internodes rather than jumping in value from one internode to the next. This result is surprising and encouraging, because MOMA simply assumes that the fluxes undergo a minimal re-distribution when the pathway system is perturbed. Because these perturbations occur independently for each internode, there is no mathematical guarantee that individual fluxes would follow any smooth trend from internode to internode. In other words, the collective results, while fitting into the context of a gradual change in lignification pattern during stem development, are by no means “automatic,” because no external constraints or conditions were imposed or enforced on the transition from one internode to the next. The computed trends are summarized in Table 1.

**Table 1:** Developmental trends in flux partitioning between successive internodes.

		Transgenic Strain					
Branch Point	Flux	PAL ↓	C4H ↓	HCT ↓	C3H ↓	CCoAOMT ↓	COMT ↓
Cinnamic acid	*v*_2_	—	—	↑	↑↑	↓↓	↑↑
	*v*_22_	—	—	↓	↓↓	↑↑	↓↓
*p*-coumaroyl-CoA	*v*_4_	↓↓	↓↓	↑	↑↑	↓↓	—
	*v*_7_	↑↑	↑↑	↑↑	↑↑	↑↑	↑↑
	*v*_23_	—	—	↓	↓↓	↑↑	↓↓
Caffeoyl-CoA	*v*_10_	—	—	—	—	—	—
	*v*_11_	—	—	—	—	—	—
Coniferyl aldehyde	*v*_14_	↓↓	↓↓	—	—	↓↓	—
	*v*_16_	↑↑	↑↑	—	—	↑↑	—
Coniferyl alcohol	*v*_15_	↓↓	↓	↓↓	↓↓	↓↓	↑
	*v*_20_	↑↑	↑	↑↑	↑↑	↑↑	↓
5-hydroxyconiferyl alcohol	*v*_21_	—	—	↓↓	—	—	—
	*v*_24_	—	—	↑↑	—	—	—

The developmental evolution of fluxes diverging at the intermediate metabolite listed in the first column, when normalized by the total flux entering the branch point, can be described as monotonically increasing (↑↑), increasing with minor variations (↑), essentially unchanged (—), decreasing with minor variations (↓), or monotonically decreasing (↓↓).

The following paragraphs are structured as follows. First, we re-evaluate the gene knock-down data in a systematic way across different stages of growth and formulate four postulates that actually do not require a full model analysis, but emerge from the “logic” of the pathway. Second, we discuss two postulates regarding novel mechanisms of metabolic regulation that result from our comprehensive model analysis. Third, we present new experimental results that directly support one of the model-based postulates.

### Availability of phenylalanine drives lignin production

The total lignin production is driven by the availability of phenylalanine rather than by enzymatic limitations. This conclusion results from the observation that the down-regulation of PAL has much less effect on total lignin content and/or lignin composition in young internodes with small amounts of lignin than in mature internodes with high lignin production (Table S3 in Text S1; [6]). Expressed differently, PAL is not acting at capacity when the demand for lignin is relatively low, as is the case in young internodes. This conclusion is also supported by the observation that lignin production is not enhanced proportionately when PAL enzyme is over-expressed in transgenic plants [21].

### HCT is reversible

**Figure 4:**
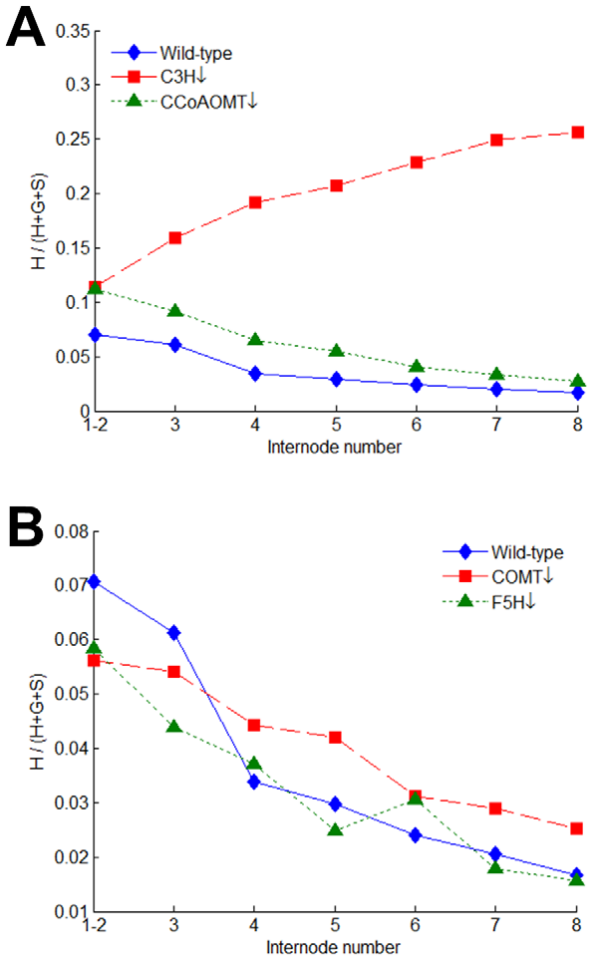
Developmental patterns of the proportion of H lignin in control and transgenic plants. (A) The proportion of H lignin in total monomer yields (H+G+S) is substantially or slightly increased in transgenic plants with reduced activities of C3H or CCoAOMT, respectively. (B) Down-regulation of COMT or F5H has essentially no effect on the proportion of H lignin in total monomer yields: the amounts of H lignin are very small and the trends do not differ from wild type.

In transgenic plants where C3H is down-regulated, the proportion of H lignin among total monomer yields is significantly increased over control plants, especially in mature internodes (Figure 4A). This finding is at first puzzling, because it is unlikely that the cell can detect changes in C3H activity and adapt accordingly by exerting appropriate flux control at an earlier branch point (*i.e.*, *p*-coumaroyl-CoA) within the network. Arguably the simplest explanation is that HCT (possibly along with other plant acyltransferases) is reversible [22]. If so, the following scenario is possible: as *p*-coumaroyl-shikimate accumulates due to a reduced C3H activity, HCT converts it back to *p*-coumaroyl-CoA in the presence of free CoA, thereby allowing the cell to escalate the production of H lignin beyond the wild-type level. The catalytic efficiency of HCT acting on *p*-coumaroyl-shikimate as substrate remains to be experimentally determined, along with the possible competition for CoA between two shikimate esters (i.e., *p*-coumaroyl-shikimate and caffeoyl-shikimate).

### C3H is mildly reversible?

The hypothesis of HCT being reversible prompts us to investigate whether C3H, which controls the material flow between two HCT-catalyzed steps, also permits catalysis in both directions. A slightly increased proportion of H lignin in CCoAOMT-deficient plants (Figure 4A) seems to suggest that C3H is mildly reversible and that part of the accumulated caffeoyl-CoA is therefore converted back to *p*-coumaroyl-CoA and subsequently channeled towards H lignin, a scenario which seems unlikely based on the known catalysis by cytochrome P450 enzymes. However, the amounts of H lignin determined by thioacidolysis appear to be unaffected by the low CCoAOMT activity despite a noticeable decrease in total lignin content (Table S3 in Text S1; [6]). One plausible explanation is that thioacidolysis yields are highly correlated with the *in vivo* abundance of S lignin [5], which might suggest that plants may in effect produce more H lignin than was measured against the down-regulation of CCoAOMT.

### Two CCR-catalyzed reactions are essentially irreversible

If both HCT and C3H are reversible, the two CCR-catalyzed reactions—*v*_10_ and *v*_13_—can be regarded as the “committed” steps (*i.e.*, they are essentially irreversible), because manipulation of any downstream enzyme, such as COMT and F5H, has no substantial effect on H lignin (Figure 4B). Interestingly, the postulate seems to echo the conclusion from a previous enzyme assay [23]: CCR purified from poplar stems was able to catalyze the conversion of coniferaldehyde into feruloyl-CoA in the presence of other co-factors but preferentially reduced CoA-esters, as judged by the calculated equilibrium constants.

### The pathway contains crossing channels towards G and S lignin

**Figure 5:**
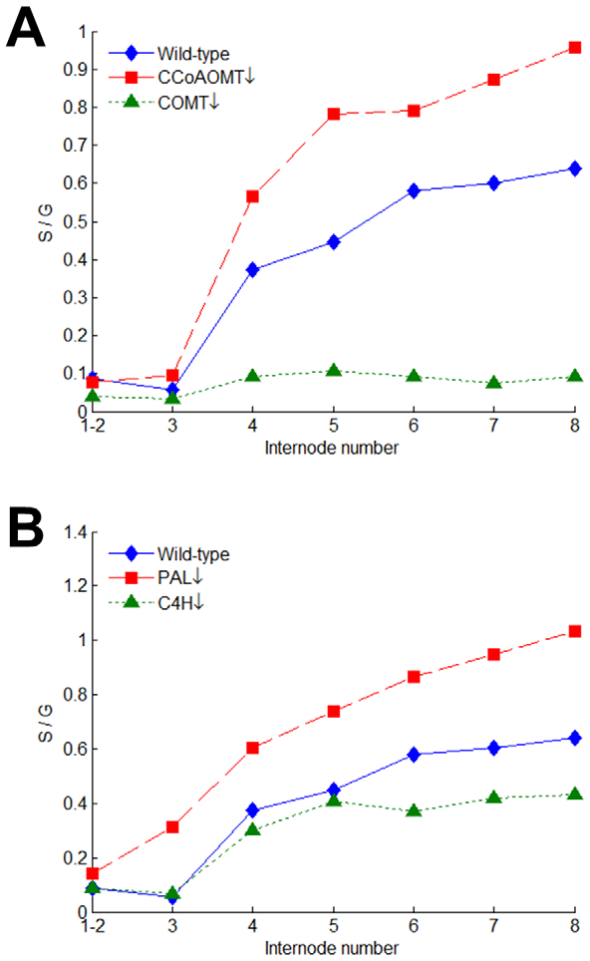
Developmental patterns of the S/G ratio in control and transgenic plants. (A) In comparison with control plants, the S/G ratio is increased in CCoAOMT-deficient plants but drastically decreased in COMT-deficient plants. (B) Similarly, the S/G ratio is increased in PAL-deficient plants but decreased in C4H-deficient plants.

In addition to a modest increase in H lignin, down-regulation of CCoAOMT leads to a noticeable increase in the S/G ratio of all internodes except for internodes 1 and 2 (Figure 5A). This finding is puzzling because coniferyl aldehyde is a common precursor to both S and G lignin and one would therefore expect a similar effect on both. The analogous situation arises in COMT-deficient plants, where the S/G ratio is reduced (Figure 5A). This case, however, is not quite as clear-cut because COMT also shows activities towards downstream intermediates like 5-hydroxyconiferyl aldehyde and 5-hydroxyconiferyl alcohol. Thus, in this case of COMT deficiency, the S/G ratio might not be a good indicator of the flux partitioning at coniferyl aldehyde towards G and S lignin.

As an explanation for the altered S/G ratios in cases of CCoAOMT or COMT down-regulation, we postulate that the enzymes controlling *v*_12_ and *v*_16_ (and maybe even *v*_10_ and *v*_17_) are organized into a functional complex through which the intermediates are channeled without much leakage. Similarly, we postulate that *v*_13_ and *v*_14_ form a corresponding complex without much leakage. This dual postulate for crossing channels is supported indirectly by literature information and by findings from our flux analysis, as outlined below.

First, an analysis of mature stems (internodes 6–9) collected from CCoAOMT down-regulated transgenic lines indicated that the levels of G lignin were greatly reduced, whereas those of S lignin were nearly unaffected (*cf.* CCOMT antisense line ACC305 in Table 1 of [24]). Similarly, down-regulation of CCR1, which actively catalyzes the subsequent reduction of feruloyl-CoA to coniferyl aldehyde, also resulted in an increased S/G ratio in mature internodes of alfalfa stems [25], again with G lignin being more strongly reduced than S lignin. Although the existence of the CCR2-COMT pathway helps sustain the lignin content in either CCoAOMT or CCR1 down-regulated lines, the findings do not explain why S lignin is synthesized at the expense of G lignin upon genetic modifications of the CCoAOMT-CCR1 pathway. Nevertheless, the findings are entirely consistent with the postulate of crossing channels.

Second, one of the constituent enzymes, F5H, is localized to the external surface of the endoplasmic reticulum [26], so that the proposed channel may exist in the form of an enzyme complex anchored in the endomembrane. Indeed, a labeling experiment in microsomes extracted from lignifying alfalfa stems suggested such a co-localization of COMT and F5H [27]. It showed that caffeyl aldehyde, when incubated with [methyl-^14^C]-labeled S-adenosyl L-methionine (a co-substrate necessary for COMT-mediated O-methylation) and NADPH (the reducing agent for F5H), is converted to coniferyl aldehyde, 5-hydroxyconiferyl aldehyde, and a small amount of sinapyl aldehyde.

**Figure 6:**
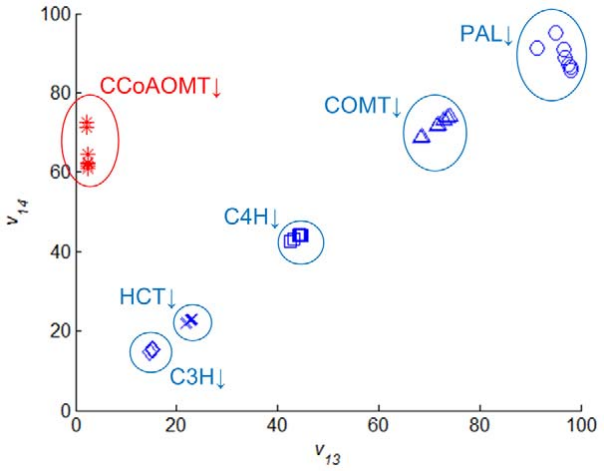
Plot of *v*_14_ versus *v*_13_ in transgenic plants. Expression of PAL, C4H, HCT, C3H, CCoAOMT, or COMT was independently down-regulated. Symbols within each ellipse represent different internodes. With the exception of CCoAOMT, the two fluxes are very strongly and linearly correlated.

Finally, our flux distribution analysis reveals a strong correlation between the computed flux values of *v*_13_ and *v*_14_ for all but the CCoAOMT-deficient plants (Pearson correlation coefficient ρ = 0.9952; *p*-value<0.001) (Figure 6). This correlation suggests that there is normally almost no exchange of products between *v*_12_ and *v*_13_, and that most of the coniferyl aldehydes produced through the CCR2-COMT shunt are directly utilized by F5H without having the opportunity of diversion into G lignin biosynthesis. A notable exception seems to be the situation where CCoAOMT is significantly down-regulated. In this case, caffeoyl-CoA tends to accumulate at least in the short term, thus providing the CCR2-COMT pathway and the associated metabolic channel with an abundance of substrate. The predicted flux distribution (Figure 3) and the observed lignin composition (Table S3 in Text S1) indicate that CCoAOMT-deficient plants produce a considerable amount of G lignin, although the levels of S lignin are comparable to those in the controls, which implies that only some of the extra caffeoyl-CoA can be converted efficiently into S lignin through the proposed channel. Overall, the proposed functional channels seem to be consistent with results of the flux analysis as well as with earlier discussions in the literature [8], [9]. The correlation between *v*_12_ and *v*_16_ is less pronounced, which is presumably due to the fact that F5H and COMT catalyze parallel pathways, with the latter (*v*_20_ and *v*_21_) buffering changes in earlier precursors.

An alternative explanation for an increased S/G ratio upon modifications of the CCoAOMT-CCR1 pathway could be that the kinetic features of the enzymes that catalyze coniferyl aldehyde and coniferyl alcohol are fine-tuned such that they could permit the adjustment of fluxes leading to G and S lignin and thus change the S/G ratio. For instance, given that down-regulation of CCoAOMT or CCR1 may alter the intracellular level of coniferyl aldehyde, the relative values of *v*_14_ and *v*_16_ at steady state could depend on whether the respective enzyme works within the linear or saturation region of its kinetic profile.

To investigate this alternate hypothesis, we designed and analyzed a kinetic Michaelis-Menten model that contains the two alternative pathways from caffeoyl-CoA to coniferyl aldehyde as well as the two principal branch points where the fluxes leading to G and S lignin diverge (see Text S1). The model was simulated 10,000 times with randomly sampled kinetic parameter values, as described in *Materials and Methods* and Text S1, and we recorded the percentage of admissible parameter sets that yielded a significantly increased S/G ratio in response to a 80% reduced CCoAOMT or CCR1 activity.

We first examined the case where CCoAOMT is down-regulated. Only ∼5% of all admissible systems (see Text S1 for definition) yielded a significantly increased S/G ratio, whereas nearly half of all systems resulted in an S/G ratio that differed by less than 5%. The few cases of significant increases in the S/G ratio did not reveal particular patterns, which may not be too surprising because the system involves 16 kinetic parameters that affect each other in a nonlinear fashion. Intriguingly, for the scenario of CCR1 down-regulation, none of the admissible systems showed a significant increase in S/G ratio; in fact, all changes in S/G ratios were less than 0.5%. Replacing the Michaelis-Menten kinetics with cooperative Hill kinetics allowed more flexibility. Still, only ∼3% of all admissible systems exhibited an increase in S/G ratio upon CCR1 down-regulation. Taken together, it seems that, theoretically, some precisely tuned sets of kinetic parameters could lead to the observed effects on the S/G ratio. However, these sets are rare and do not seem to be robust enough to render the kinetics-based hypothesis viable.

### Feedforward regulation by a compound derived from cinnamic acid

One of the most paradoxical findings among the collective results from the transgenic plants is the opposite effect on lignin composition (and specifically the S/G ratio) when either PAL or C4H is down-regulated. It seems that these alterations should not differentially affect monolignol biosynthesis, because both occur before the first branch point, but they do. Closer inspection of the data from different internodes reveals that the S/G ratio is consistently increased in PAL-deficient plants but decreased in C4H-deficient plants (Figure 5B). While experiments with tobacco have suggested that the differential co-localization of PAL isoforms and C4H might be the underlying cause of such observations [28], there is as yet no direct evidence for this intracellular association in alfalfa or other related legume species.

In accordance with the proposition of separate metabolic channels for G and S lignin, we postulate that the different effects of PAL or C4H down-regulation on the S/G ratio are due to feedforward regulation. Specifically, we suggest that this regulation is mediated by a downstream product of the cinnamic acid degradation pathway, which is represented collectively as *v*_22_ in Figure 1. Notice that this feedforward regulation had not been recognized by the scientific community and was postulated by the model analysis purely with computational means.

**Figure 7:**
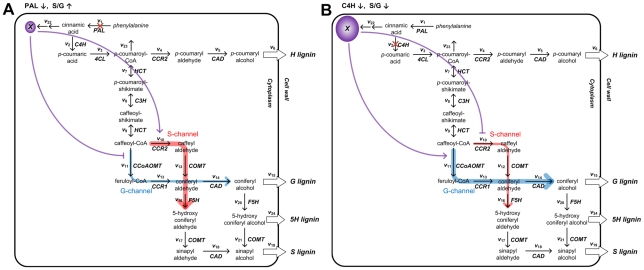
Effects of PAL (A) or C4H (B) down-regulation on the postulated channels. The postulated G lignin- and S lignin-specific channels are colored in blue and red, respectively, with their widths representing the relative capacity in the designated transgenic plants. The size of the circle with the unknown compound *X* correlates symbolically with its intracellular pool size. The blocked purple line indicates repression, whereas the purple arrow indicates activation.

**Figure 8:**
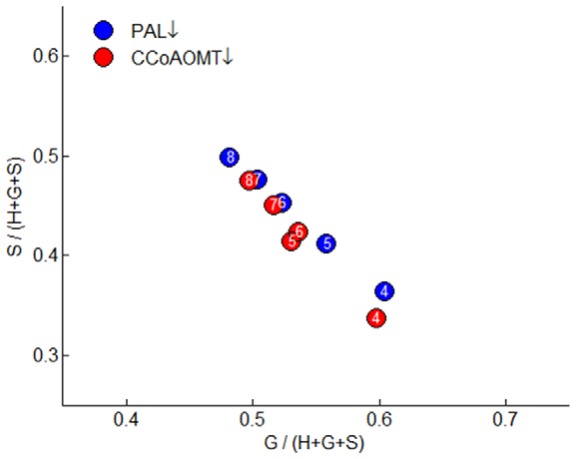
Plot of the proportion of S versus the proportion of G in total monomer yields. The colored circles represent internodes 4–8 of stems collected from transgenic plants where PAL or CCoAOMT is down-regulated. Numbers in the symbols refer to the specific internodes.

Consistent with the observation of all transgenic experiments, an appropriate control strategy by this unknown compound X is summarized in Figure 7 and discussed below. In the case of PAL-deficiency, where the biosynthesis of cinnamic acid from phenylalanine declines, a diminished pool of X could directly or indirectly reduce the expression of CCoAOMT/CCR1/CAD and/or activate the expression of CCR2/COMT/F5H, thereby altering the channeling towards G and S lignin and increasing the S/G ratio. Intriguingly, this proposed inhibition of CCoAOMT expression following PAL down-regulation is supported by a strong correlation of the proportion of G and S lignin in total monomer yields in internodes 4–8 of the PAL- and CCoAOMT-deficient plants (Figure 8).

In the case of C4H deficiency, however, the production of X through *v*_22_ is likely to increase because the consumption of cinnamic acid through a competing branch *v*_2_ is not as effective as in wild-type plants. Thus, an accumulation of X could in turn activate the expression of CCoAOMT/CCR1/CAD and/or reduce the expression of CCR2/COMT/F5H, leading to a smaller S/G ratio.

### Salicylic acid is a signaling molecule for monolignol biosynthesis

**Figure 9:**
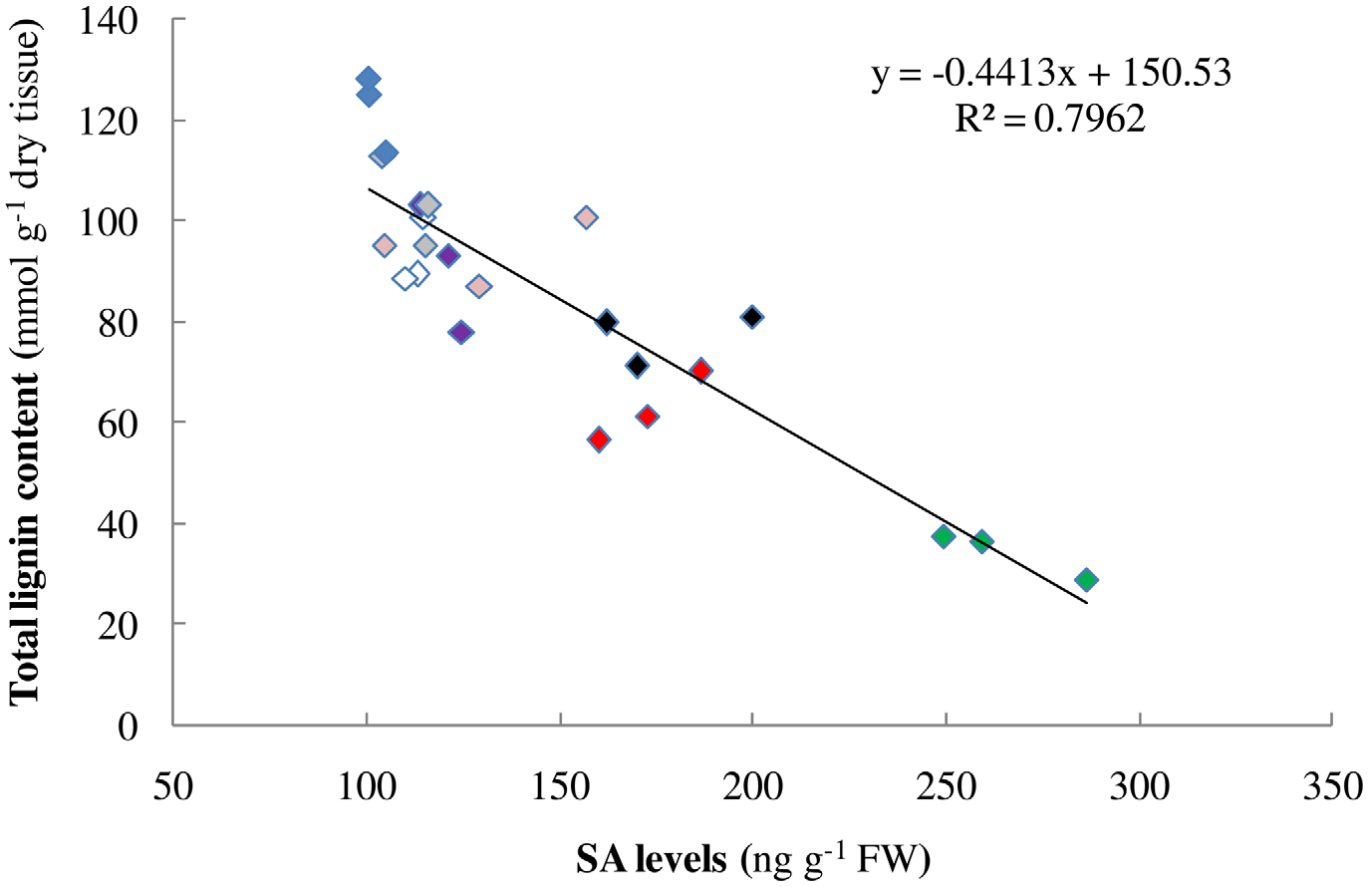
Relationship between lignin content and salicylic acid accumulation in different alfalfa antisense lignin down-regulated lines.

Salicylic acid (SA) is a notable endogenous signaling molecule that is known to be derived from cinnamic acid [29]. Down-regulation of one pathway enzyme other than C4H (*e.g.* HCT [30]) had recently been shown to lead to elevated levels of SA. To investigate whether SA is the postulated signaling compound X, we measured its intracellular levels in many independent transgenic alfalfa lines in which different monolignol biosynthesis genes had been down-regulated. Indeed, the results show that the intracellular levels of SA are highly proportional to the extent of lignin reduction (Figure 9). Based on our postulated feedforward regulation, this effect can be explained through the participation of SA in the inhibition of the metabolic channel committed to S lignin biosynthesis, thus reducing the total lignin content.

## Discussion

Functional genomics is a premier tool for identifying metabolic pathways in sequenced model species and for pinpointing genes involved in them [31]. However, it is known that many enzymes coexist in multiple isoforms with unique expression patterns and substrate specificities. A pertinent example seems to be the recent discovery of two CCR isoforms with distinct catalytic properties towards major CoA-esters in *Medicago* [7]. Steady-state flux analysis of an extended pathway system that accounts for the isoforms reveals that the alternative path is dispensable in wild-type plants, but that it may rise to significant levels in specific transgenic lines. Indeed, CCoAOMT-deficient plants support a much higher lignin production than lines where HCT or C3H is down-regulated (Table S3 in Text S1; [6]). The intricate differences in pathway operation among otherwise very similar transgenic lines point to the need of investigating flux patterns not only in different plants, but also in different strains, lines and even different internodes and tissues. The results shown here furthermore demonstrate that subtle variances among tissues and lines are difficult to discern with intuition alone, but that computational analyses can serve as objective and rigorous tools for explaining such differences.

Specifically, the new integrative modeling approach proposed here combines static flux-based models and a Monte Carlo simulation of randomly parameterized kinetic models. This approach has the advantage that it allows the collective analysis of many experimental results and sheds light on pathway features that are particularly important for functionality under normal and altered conditions. The analysis here revealed a quantitative trend of flux patterns during development, which in turn allowed the identification of principal branch-point metabolites at which internode-specific flux partitioning patterns control the observed mode of lignification. While it is relatively easy to single out principal metabolites in linear or slightly branched pathways, the system studied here is confounded by the plant's employment of the same enzymes, such as CCR and CAD, in different key positions. Due to this multiple use, manipulating the flux partitioning pattern towards a desired mode of lignification may incur undesired “side effects.”

The computational analysis indicates that a single flux analysis just for wild-type plants is insufficient for understanding because even a seemingly simple pathway like monolignol biosynthesis requires relatively minor, yet important, extensions to account for the overflow of some intermediate metabolites that only occurs in transgenic plants. At the same time, the analysis also demonstrates that the simultaneous analysis of several independent datasets, in this case transgenic lines and sequential internodes, can lead to insights that otherwise would have been difficult to obtain. Here, it led to several postulates that are specific enough for experimental validation or refutation.

Some model-free postulates refer to the need for reversibility or committedness of key reactions, which might not be too surprising. Two further postulates are more intriguing. They refer to the functional channeling within the pathway and its mechanistic control. Based on the observation of an increased S/G ratio in CCoAOMT or CCR1 down-regulated lines, the computational results suggest an S lignin-specific channel capable of converting caffeyl aldehyde directly into 5-hydroxyconiferyl aldehyde or sinapyl aldehyde. Different experiments in the literature suggested the co-localization of COMT and F5H in lignifying alfalfa stems [27] and the localization of F5H to the external surface of the endoplasmic reticulum [26]. These and our findings would imply the likely location for a functional S-channel complex to be associated with the endomembrane.

While the proposed membrane-bound channel for synthesizing S lignin could constitute an important control mechanism, it may only have comparatively limited capacity because even in CCoAOMT down-regulated lines G lignin is generated in a higher proportion of total monomer yields than S lignin (Table S3 in Text S1; [6]). One likely cause is that different *O*-methyltransferases (OMTs) are involved in converting caffeyl aldehyde to coniferyl aldehyde. These OMTs may have distinct sub-cellular localization (to cytoplasm or endomembrane) and therefore a different affinity to F5H. Thus, it could be that the cytosolic OMT in the transgenic lines with reduced CCoAOMT expression is up-regulated and helps consume extra caffeyl aldehyde outside the proposed channel. A corresponding labeling experiment in alfalfa [27] confirmed that only a small proportion of total cellular COMT activity against caffeyl aldehyde is associated with the microsomal membrane, and that adding excess recombinant COMT has little effect on the metabolism of caffeyl aldehyde by microsomes.

To examine whether the observed increase in the S/G ratio upon modifications of the CCoAOMT-CCR1 pathway could be explained alternatively by a kinetically-controlled mechanism, we generated 10,000 ODE models for a reduced pathway system (Text S1) and simulated both down-regulation schemes. Among all sampled parameter sets, only a minute percentage of systems had the ability to increase their S/G ratio significantly in either case. Although the results neither reject the possibility of a kinetically-controlled S/G ratio nor directly corroborate our channeling postulate, they do suggest that purely kinetic control might be unlikely, because it would require rather precise implementations of specific parameters in different tissues, which seems to compromise the robustness of the system. As shown in a structural study of alfalfa COMT [32], mutations of some key residues lining the active site result in significantly different substrate binding and/or turnover rate. Moreover, it is likely that the kinetic properties of other enzymes may also exhibit a similar, if not more severe, susceptibility to genetic perturbations (e.g., [33], [34]). Since the variation in the S/G ratio is typically small (s.d.≈0.03 in two control lines; [6]), the proposed functional channeling mechanisms seem to offer a more robust option to help maintain a physiologically proper S/G ratio.

The observed decrease in the S/G ratio of COMT down-regulated lines alone is not sufficient to prove the existence of a G lignin-specific channel, because a reduced COMT activity affects all fluxes that are specific for the synthesis of S lignin, thus leading to a smaller S/G ratio. Nevertheless, the strong correlation between *v*_13_ and *v*_14_ that emerged from our computations for most transgenic experiments lends further credence to such an inference. This correlation not only supports the operation of a G lignin-specific channel, but also hints at the possibility of CCR1 and CAD (and maybe CCoAOMT) being complexed or co-localized on internal membranes.

One option for testing this postulate would be to down-regulate CCR2 and record if the strain exhibits a greater decrease in S lignin than in G lignin, giving a smaller S/G ratio. Surprisingly, knocking out CCR2 in *M. truncatula*, a species closely related to alfalfa, leads to an increased S/G ratio, whereas *M. truncatula* CCR1 knock-out mutants show a reduction in the S/G ratio [7]. However, in spite of their close taxonomic relatedness, the operation and control of monolignol biosynthesis might be quite different in tetraploid alfalfa (*M. sativa* L.) and diploid *M. truncatula*. For instance, the S/G ratio in wild-type alfalfa stems (0.62; internodes 1–8) is approximately twice as large as that in wild-type *M. truncatula* stems (0.29; internodes 1–7). Consequently, further experimental work is required to validate or reject the postulate that a G lignin-specific channel is operational in alfalfa.

If the postulates of specific channels towards the synthesis of G and S lignin are valid, one may further surmise that the opposite effects of PAL or C4H down-regulation on lignin composition are the results of differential gene or enzyme expression, which could be mediated by a cinnamic acid derivative. However, the model could not identify this molecule, leading us to call it *Compound X*. Supporting this hypothesis, the transgenic experiments used here have shown that down-regulation of CCoAOMT, which we postulate to be involved in the G lignin-specific channel, yields similar proportions of G and S lignin among total monomers as does the down-regulation of PAL, which is postulated to inhibit and/or activate the functioning of the G lignin- and S lignin-specific channels, respectively (Figure 8).

**Figure 10:**
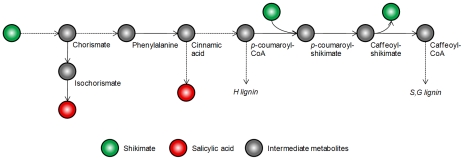
Alternative routes of salicylic acid biosynthesis and shikimate recycling. Steps likely to involve more than one enzyme and intermediate are shown with dashed arrows. In addition, pathways such as the tyrosine and flavonoid branches are not shown for the sake of clarity.

Salicylic acid (SA), a phenolic phytohormone derived from phenylalanine, was proposed as a potential candidate for this unknown Compound X. Intriguingly, post-hoc experiments showed that the intracellular levels of SA are indeed highly proportional to the extent of lignin reduction in transgenics where different pathway genes are down-regulated (Figure 9). This result fits directly into the context of our feedforward control postulate. At the same time, it makes us wonder why putting a block on monolignol biosynthesis could affect the homeostasis of SA, especially if the blockage is located away from the pathway entrance. Based on previous findings that SA can be derived both from cinnamic acid and from isochorismate via the shikimate pathway [29], and that HCT uses shikimate as a preferred cofactor (Figure 10), we propose the following scenario: when the flux going through the pathway is decreased due to some genetic manipulation, fewer shikimates will be trapped in those shikimate esters (*p*-coumaroyl-shikimate and caffeoyl-shikimate) and thus become available to make SA. In other words, the shikimate recycling facilitated by HCT enables the shikimate pool to work as a sensor of the flux into lignin. Future in-depth studies, whether they are experimental or computational, are required to justify this hypothesis. It is noteworthy, however, that the reason why plants shuttle monolignol pathway intermediates between Coenyme A and shikimate esters has yet to be explained.

In conclusion, our analysis shows that a combined modeling effort can uniquely and effectively complement experimental studies of the type used here. In contrast to analyzing one dataset at a time, it allowed us to integrate all results from a comprehensive experimental investigation of various transgenic lines and internodes. This integration, in turn, revealed dynamic, developmental patterns and their dependence on key enzymes. Together, the analyses uncovered elusive control of monolignol biosynthesis and led to testable hypotheses regarding various pathway aspects that should be clarified before one attempts to generate and optimize viable, productive “designer” crops with minimal recalcitrance.

## Materials and Methods

### Experimental data

In a previous study [6], lignin content and composition were analyzed in transgenic alfalfa plants in which seven enzymes were independently down-regulated (*cf.* Figure 1). These enzymes were: L-phenylalanine ammonia-lyase (PAL), cinnamate 4-hydroxylase (C4H), hydroxycinnamoyl CoA:quinate/shikimate hydroxycinnamoyl transferase (HCT), coumarate 3-hydroxylase (C3H), caffeoyl coenzyme A 3-*O*-methyltransferase (CCoAOMT), ferulate 5-hydroxylase (F5H), and caffeic acid 3-*O*-methyltransferase (COMT). Each transgenic plant was cultivated to early flowering stage, and the mature stem consisting of eight internodes was harvested and divided into individual segments; all internodes were numbered according to their maturity, with internodes 1–2 representing the pooling of the two uppermost stem segments. The lignin content and monomer composition for each internode were determined for each transgenic line via established protocols [6]; the results are summarized in Table S3 in Text S1. The activities of all targeted enzymes were also measured and summarized elsewhere, with the exception of F5H, which showed no activity towards any documented substrates when assayed in crude alfalfa extracts *in vitro* (Table 2c of [6]). Thus, the F5H-deficient line is excluded from the following analysis.

#### Salicylic acid determination

Salicylic acid levels in stems from the same plant lines (excepting PAL down-regulated plants) as well as from CAD down-regulated lines [25] were determined using the biosensor *Acinetobacter* sp. ADPWH_lux as described previously [35], [36]. Samples consisted of detached stems consisting of six internodes. SA was extracted by grinding stems (100 mg fresh weight) in fresh LB liquid medium (2.5 ml LB per 1 g of stem) by vortexing for 30 sec and sonicating for 5 min on ice, after which the homogenates were centrifuged at 12,000 g for 15 min. The supernatants were used for SA measurement and an equivalent volume of LB medium was used to make a SA standard curve (SA final concentrations of 0, 0.05, 0.25, 0.5, 1.6, 8.3, 20, 40, 83, 166 and 200 µM). An overnight culture of *Acinetobacter* sp. ADPWH_lux was diluted in LB (1∶20) and grown at 37°C for ∼2 hrs to an OD_600_ of 0.4. Sixty µl of LB medium, 50 µl of salicylate biosensor culture and 20 µl of each crude extract were mixed in a 96-well cell culture plate. The plate was incubated at 37°C for 1 h without shaking and bioluminescence and OD_600_ of negative controls (LB alone or water) were read using a Glomax Multi detection system (Promega Corporation, Sunnyvale, CA). SA standard and negative controls were read in parallel with the experimental samples and every sample was replicated five times. Relative bioluminescence was obtained by subtracting bioluminescence OD_600_ of negative controls, and SA concentration was estimate according to the SA standard curve.

#### Lignin content

Lignin content was determined by the thioacidolysis method as described previously [30].

### Modeling approach

#### FBA and MOMA

The static flux balance models build on the assumption that the metabolic pathway system is in a quasi-steady state where, for any metabolite pool, fluxes governing its synthesis and degradation are equal. Mathematically, such a mass balance constraint can be represented as
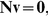
(1)where **N** is the stoichiometric matrix of the pathway system and **v** is a vector of fluxes. Other commonly used constraints are the upper and lower bounds on individual fluxes
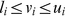
(2)that define the possibility of reversibility and the maximal reaction rate, respectively. Here, we assume that all the metabolic reactions and transport processes are irreversible and therefore set 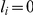 for all *i*. The only exceptions are the three overflow fluxes (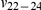; defined in *Results*), for which we arbitrarily choose 0.01 as the lower bound to prevent their values from becoming too small in the subsequent optimization step. The maximal reaction rates used for defining the upper bounds are currently unavailable because most enzymes within the pathway have not been characterized in *Medicago*, according to the enzyme databases like BRENDA [37]. Therefore, we normalized all fluxes to the value of 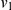 as a means of standardization. This normalization, which is achieved by introducing an extra constraint 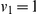, works to ensure that all fluxes are less than or equal to one.

Specific to this work, the measured, relative amounts of lignin monomers can be reformulated as “proportionality constraints” on the three fluxes 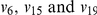 that represent the transport of monolignols into the cell wall (Figure 1). As an illustration, if the first two stem internodes consist of 7% H lignin, 85.5% G lignin, and 7.4% S lignin, we can define the following equality constraints:
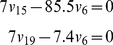
(3)For a specific internode of wild-type alfalfa plants, all constraints taken together define the feasible space of all permissible flux distribution, which is denoted by 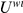. In FBA, the optimal solution is identified within 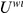 by solving a linear programming problem where an appropriate objective function *f* is maximized or minimized. In the case of lignin biosynthesis, we assume that the lignified stem tissues of *wild-type* alfalfa plants have evolved to maximize the production of lignin monomers, which translates into the following objective function
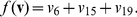
(4)The optimal flux distribution in wild-type alfalfa plants, resulting from this maximization, is denoted as 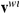.

The issue of multiple solutions giving the same optimal value of the objective function has been widely discussed [38], [39], [40]. In contrast to genome-scale models, we have the opportunity here to enumerate all equivalent flux distributions for a moderately-sized metabolic pathway system like monolignol biosynthesis, for instance, using Gauss-Jordan elimination. This advantage in turn enables us to identify a unique, physiologically relevant flux distribution for wild-type plants (see Text S1 for further details).

For lignin-modified lines, where a particular enzyme is genetically down-regulated, we use the *method of minimization of metabolic adjustment* (MOMA) [12] to predict their altered flux distributions. In its original application to gene knockout studies in bacteria, MOMA posited that a mutant strain tries to function as similarly to the wild type as possible within the limitations imposed by the mutation. In mathematical terms, the effect of a gene knockdown on the metabolic pathway system is mimicked by imposing an extra inequality constraint 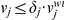 on reaction *j*: If 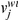 is the wild-type flux, then the activity of the mutated enzyme catalyzing this reaction is down-regulated to at most 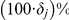 of the wild-type activity. The feasible space consisting of all flux distributions in mutants is thus defined by these inequality constraints along with all balance constraints and upper and lower bounds for the same wild-type internode, as discussed above. The notable difference for the mutant is that the specific values of the lignin monomer composition can now be significantly different (*cf.* Table S3 in Text S1). Within this reduced feasible space, which is denoted by 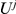, the MOMA solution 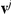 is the point that is closest to the reference point 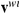 in terms of the Euclidean distance
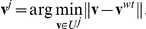



#### Monte Carlo analysis of kinetic parameters

A major surprise emerging from the experiments with lignin-modified alfalfa lines was the differential effect of CCoAOMT down-regulation on G and S lignin production. It is conceivable that these results could be due to the kinetic features of the participating enzymes. To analyze this possibility, we designed a kinetic model of the involved reactions and tested this model with a large-scale simulation. The details of this model can be found in Text S1 and the Monte Carlo techniques *per se* are straightforward. Importantly, this Monte Carlo-type simulation allowed us to examine thousands of combinations of kinetic parameters without limiting ourselves to a few particular cases of manually tuned, ill-characterized parameter values.

## Supporting Information

Figure S1 Developmental evolution of the steady-state flux distribution in PAL-deficient plants versus wild-type plants. Please refer to Figure 3 legend for explanation of boxes.
(TIF)

Figure S2 Developmental evolution of the steady-state flux distribution in C4H-deficient plants versus wild-type plants. Please refer to Figure 3 legend for explanation of boxes.
(TIF)

Figure S3 Developmental evolution of the steady-state flux distribution in HCT-deficient plants versus wild-type plants. Please refer to Figure 3 legend for explanation of boxes.
(TIF)

Figure S4 Developmental evolution of the steady-state flux distribution in C3H-deficient plants versus wild-type plants. Please refer to Figure 3 legend for explanation of boxes.
(TIF)

Figure S5 Developmental evolution of the steady-state flux distribution in COMT-deficient plants versus wild-type plants. Please refer to Figure 3 legend for explanation of boxes.
(TIF)

Text S1 Supplementary materials. This supplementary text includes two main sections. In the first section, we present the formulation of FBA and MOMA, and identify equivalent pathways that underlie the occurrence of alternate FBA solutions. In the second section, we present a kinetic model for the analysis of pathway operation at the critical branch point of coniferyl aldehyde.
(DOC)
