# Supplementary figures and images for: Integrative Analysis of Transgenic Alfalfa (Medicago sativa L.) Suggests New Metabolic Control Mechanisms for Monolignol Biosynthesis
Source: PLoS Comput Biol. 2011 May 19;7(5):e1002047. doi: 10.1371/journal.pcbi.1002047 (PMC3098223; doi:10.1371/journal.pcbi.1002047)

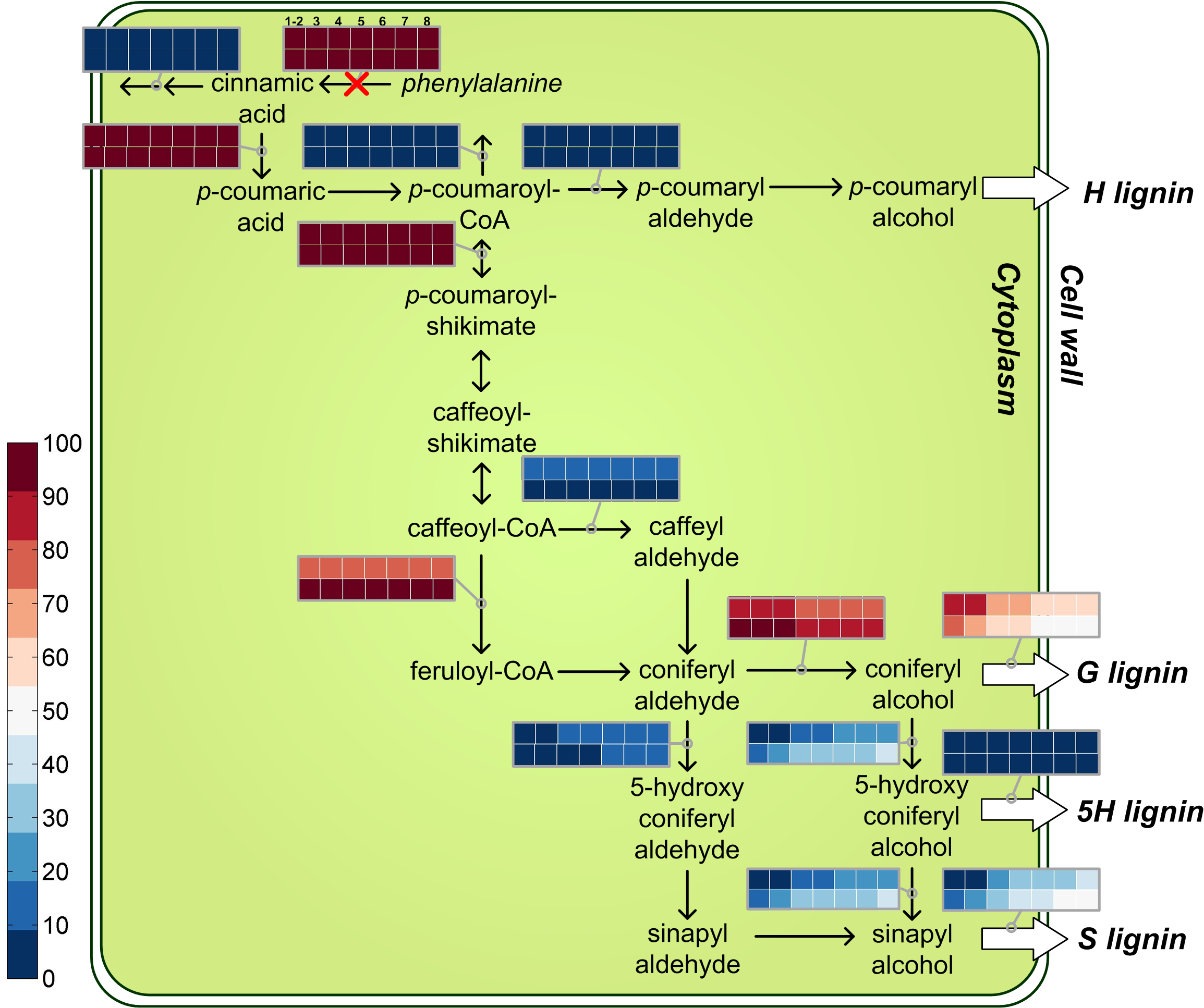

Supplement: Figure S1 — Developmental evolution of the steady-state flux distribution in PAL-deficient plants versus wild-type plants. Please refer to Figure 3 legend for explanation of boxes. (TIF) [file pcbi.1002047.s001.tif]

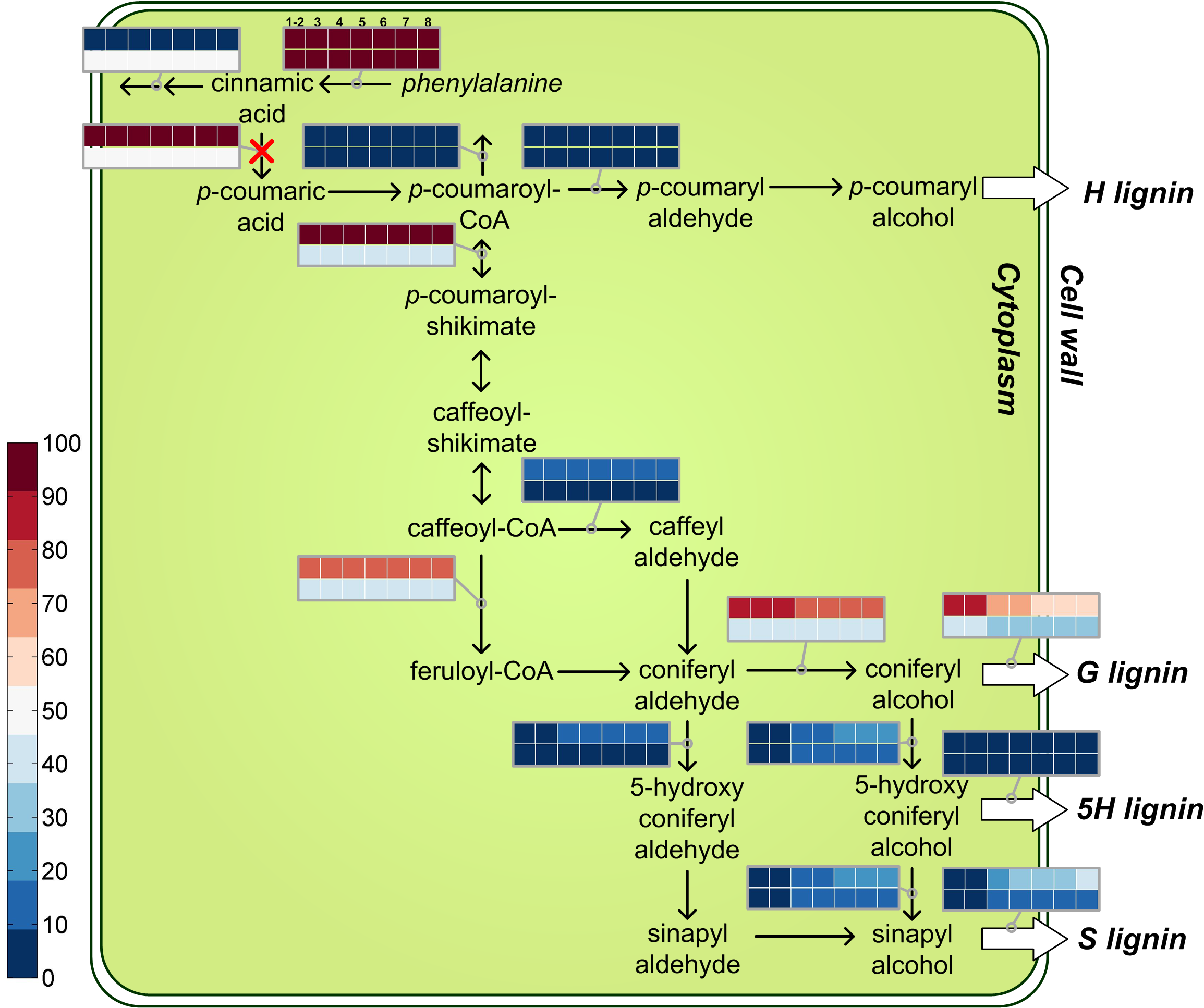

Supplement: Figure S2 — Developmental evolution of the steady-state flux distribution in C4H-deficient plants versus wild-type plants. Please refer to Figure 3 legend for explanation of boxes. (TIF) [file pcbi.1002047.s002.tif]

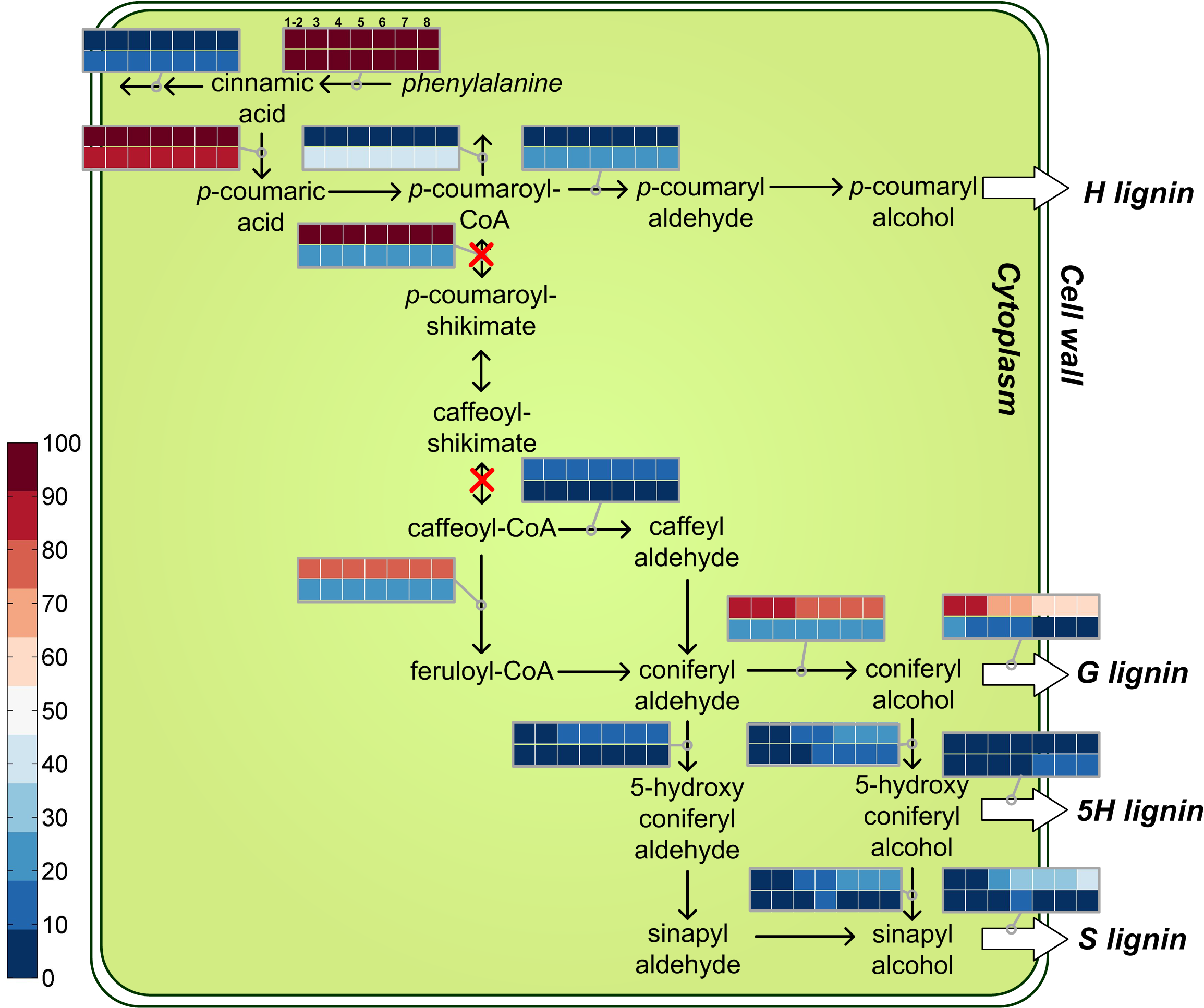

Supplement: Figure S3 — Developmental evolution of the steady-state flux distribution in HCT-deficient plants versus wild-type plants. Please refer to Figure 3 legend for explanation of boxes. (TIF) [file pcbi.1002047.s003.tif]

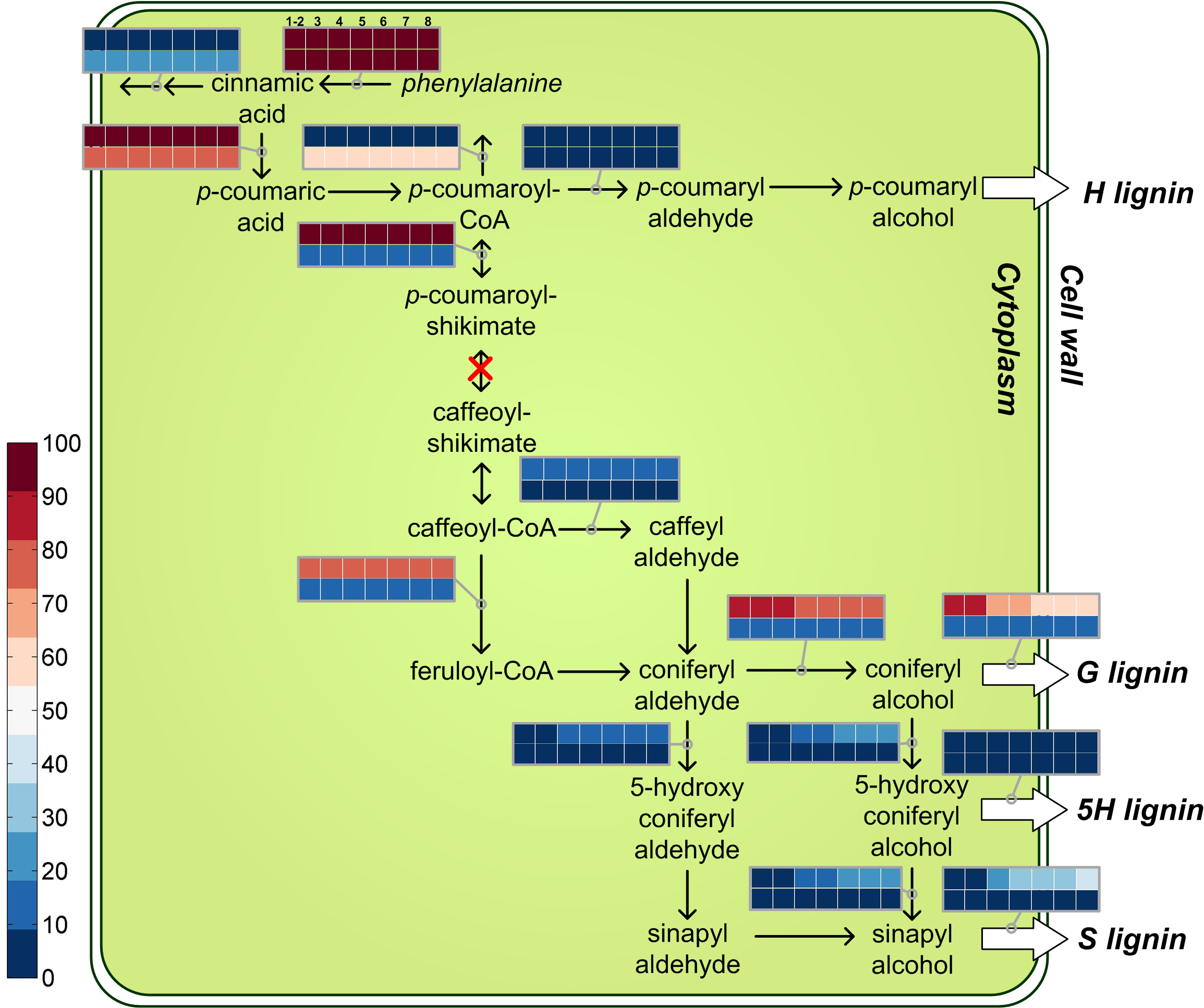

Supplement: Figure S4 — Developmental evolution of the steady-state flux distribution in C3H-deficient plants versus wild-type plants. Please refer to Figure 3 legend for explanation of boxes. (TIF) [file pcbi.1002047.s004.tif]

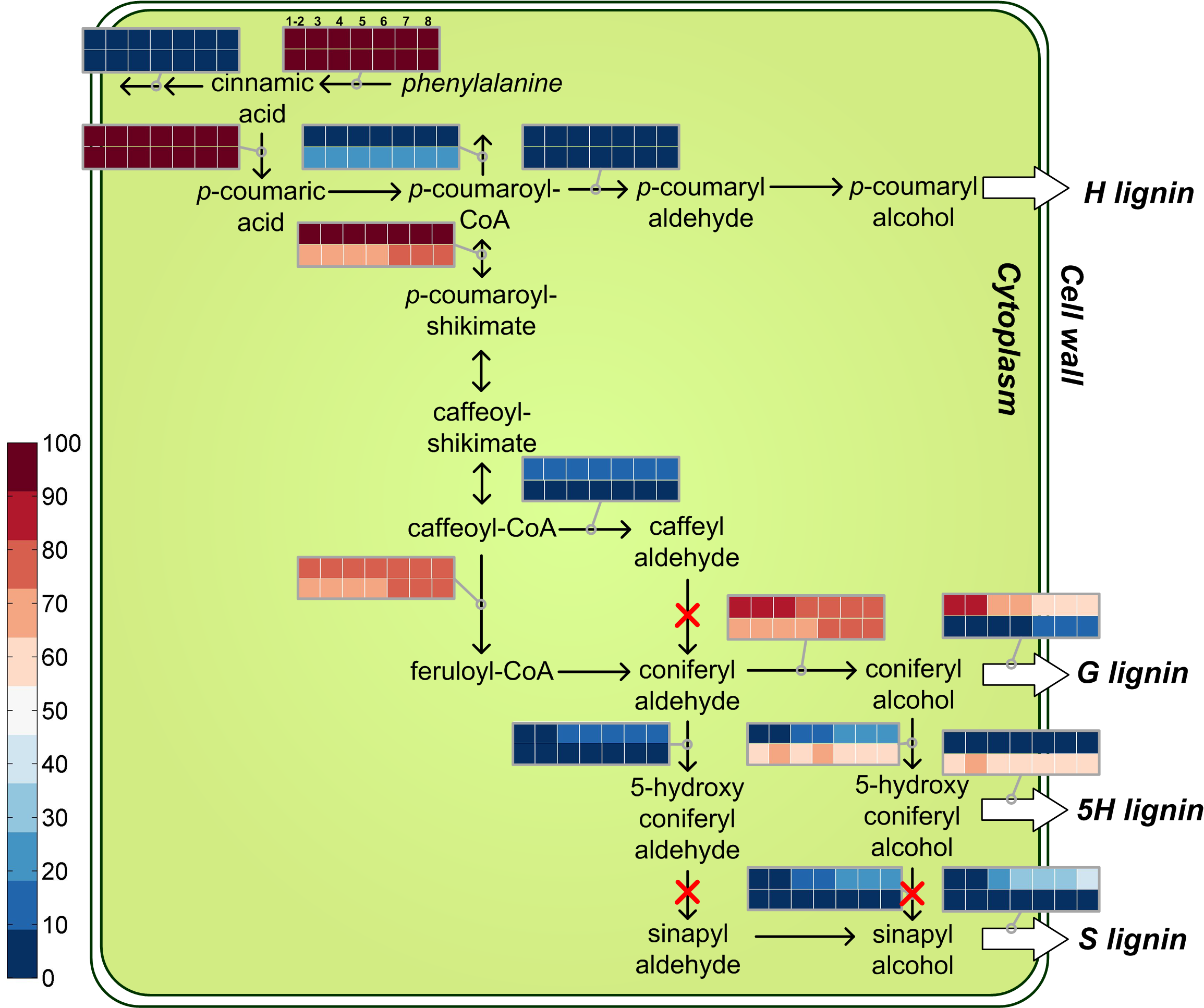

Supplement: Figure S5 — Developmental evolution of the steady-state flux distribution in COMT-deficient plants versus wild-type plants. Please refer to Figure 3 legend for explanation of boxes. (TIF) [file pcbi.1002047.s005.tif]
