# Supplementary material for: Integrative Analysis of Transgenic Alfalfa (Medicago sativa L.) Suggests New Metabolic Control Mechanisms for Monolignol Biosynthesis
Source: PLoS Comput Biol. 2011 May 19;7(5):e1002047. doi: 10.1371/journal.pcbi.1002047 (PMC3098223; doi:10.1371/journal.pcbi.1002047)
Supplement: Text S1 — Supplementary materials. This supplementary text includes two main sections. In the first section, we present the formulation of FBA and MOMA, and identify equivalent pathways that underlie the occurrence of alternate FBA solutions. In the second section, we present a kinetic model for the analysis of pathway operation at the critical branch point of coniferyl aldehyde. (DOC) [file pcbi.1002047.s006.doc]

**Text S1: Supplementary Materials**

# Integrative Analysis of Transgenic Alfalfa (*Medicago sativa* L.)

# Suggests New Metabolic Control Mechanisms for Monolignol Biosynthesis

### Yun Lee, Fang Chen, Lina Gallego-Giraldo, Richard A. Dixon and Eberhard O. Voit

This supplementary text includes three main sections. In the first section, we present the model formulation, and identify equivalent pathways that underlie the occurrence of alternate flux balance analysis (FBA) solutions. In the second section, we present a kinetic model for the analysis of pathway operation at the critical branch point of coniferyl aldehyde.

**I. Use of flux balance analysis (FBA) and minimization of metabolic adjustment (MOMA) for modeling monolignol biosynthesis**

**Model formulation**

We constructed steady-state flux-based models for wild-type and transgenic plants based on the revised pathway structure (Fig. 1 of the *Main Text*). The model comprises 24 flux variables; Table S1 shows the corresponding metabolic reaction or transport process for each flux. If a reaction is associated with a specific isozyme, as in the case of CCR1 and CCR2, the encoding *Medicago* gene (represented by its tentative consensus TC number) is also listed.

**Table S1: List of Flux Variables and Their Corresponding Metabolic R**eaction

| **Flux** | **Enzyme**  **(TC#)** | **Reaction** |
| --- | --- | --- |
| *v*_1_ | PAL | L-phenylalanine → cinnamic acid + NH_3_ |
| *v*_2_ | C4H | cinnamic acid + NADPH + O_2_ → *p*-coumaric acid + NADP^+^ + H_2_O |
| *v*_3_ | 4CL | *p*-coumaric acid + CoA + ATP → *p*-coumaroyl-CoA + Pi + AMP |
| *v*_4_ | CCR2 (TC100678) | *p*-coumaroyl-CoA + NADPH → *p*-coumaryl aldehyde + NADP^+^ + CoA |
| *v*_5_ | CAD | *p*-coumaryl aldehyde + NADPH → *p*-coumaryl alcohol + NADP^+^ |
| *v*_6_ | Tr* | *p*-coumaryl alcohol → Ø |
| *v*_7_ | HCT | *p*-coumaroyl-CoA + shikimate → *p*-coumaroyl-shikimate + CoA |
| *v*_8_ | C3H | *p*-coumaroyl-shikimate + NADPH + O_2_ → caffeoyl-shikimate + NADP^+^ + H_2_O |
| *v*_9_ | HCT | caffeoyl-shikimate + CoA → caffeoyl-CoA + shikimate |
| *v*_10_ | CCR2 (TC100678) | caffeoyl-CoA + NADPH → caffeoyl aldehyde + NADP^+^ + CoA |
| *v*_11_ | CCoAOMT | caffeoyl-CoA + *S*-adenosyl-L-methionine → feruloyl-CoA + S-adenosyl-homocysteine |
| *v*_12_ | COMT | caffeoyl aldehyde + *S*-adenosyl-L-methionine → coniferyl aldehyde + S-adenosyl-homocysteine |
| *v*_13_ | CCR1 (TC106830) | feruloyl-CoA + NADPH → coniferyl aldehyde + NADP^+^ + CoA |
| *v*_14_ | CAD | coniferyl aldehyde + NADPH → coniferyl alcohol + NADP^+^ |
| *v*_15_ | Tr | coniferyl alcohol → Ø |
| *v*_16_ | F5H | coniferyl aldehyde + NADPH + O_2_ → 5-hydroxyconiferyl aldehyde + NADP^+^ + H_2_O |
| *v*_17_ | COMT | 5-hydroxyconiferyl aldehyde + *S*-adenosyl-L-methionine → sinapyl aldehyde + S-adenosyl-homocysteine |
| *v*_18_ | CAD | sinapyl aldehyde + NADPH → sinapyl alcohol + NADP^+^ |
| *v*_19_ | Tr | sinapyl alcohol → Ø |
| *v*_20_ | F5H | coniferyl alcohol + NADPH + O_2_ → 5-hydroxyconiferyl alcohol + NADP^+^ + H_2_O |
| *v*_21_ | COMT | 5-hydroxyconiferyl alcohol + NADPH → sinapyl alcohol + NADP^+^ |
| *v*_22_ | N/A^†^ | cinnamic acid →→ salicylic acid |
| *v*_23_ | N/A^†^ | *p*-coumaroyl-CoA →→ anthocyanin, flavonoid, isoflavonoid,… |
| *v*_24_ | Tr | 5-hydroxyconiferyl alcohol → Ø |

*Tr represents collectively all biochemical events during the transport of alcohol precursors into the cell wall, *i.e.*, outside the cytoplasm (Ø).

^†^*v*_22_ and *v*_23_ refer to the sequence of reactions that leads to the synthesis of salicylic acid and flavonoid derivatives, respectively. Thus, they are not associated with a single enzyme.

Typically, two classes of constraints are employed for steady-state flux balance models. The first is conservation of mass, which can be characterized mathematically by Eq. 1 of the *Main Text*. Instead of presenting the constraint as the product of a stoichiometric matrix and a column vector of fluxes, we list the mass balance equation for each of the 16 intermediate metabolites in Table S2. Variables colored in red refer to the three “overflow” fluxes in Fig.1 of the *Main Text*. Details of the second class of constraints, which concerns the reversibility and maximal reaction rates of individual fluxes, have been discussed in the *Main Text* and will not be repeated here.

**Table S2: Mass Balance E**quations.

| **Metabolite** | **Balance Equation of Influxes and Effluxes** |
| --- | --- |
| cinnamic acid | *v*_1_ – *v*_2_ – *v*_22_* = 0 |
| *p*-coumaric acid | *v*_2_ – *v*_3_ = 0 |
| *p*-coumaroyl-CoA | *v*_3_ – *v*_4_ – *v*_7_ – *v*_23_ = 0 |
| *p*-coumaryl aldehyde | *v*_4_ – *v*_5_ = 0 |
| *p*-coumaryl alcohol | *v*_5_ – *v*_6_ = 0 |
| p-coumaroyl-shikimate | *v*_7_ – *v*_8_ = 0 |
| caffeoyl-shikimate | *v*_8_ – *v*_9_ = 0 |
| caffeoyl-CoA | *v*_9_ – *v*_10_ – *v*_11_ = 0 |
| caffeoyl aldehyde | *v*_10_ – *v*_12_ = 0 |
| feruloyl-CoA | *v*_11_ – *v*_13_ = 0 |
| coniferyl aldehyde | *v*_12_ + *v*_13_ – *v*_14_ – *v*_16_ = 0 |
| coniferyl alcohol | *v*_14_ – *v*_15_ – *v*_20_ = 0 |
| 5-hydroxyconiferyl aldehyde | *v*_16_ – *v*_17_ = 0 |
| sinapyl aldehyde | *v*_17_ – *v*_18_ = 0 |
| 5-hydroxyconiferyl alcohol | *v*_20_ – *v*_21_ – *v*_24_ = 0 |
| sinapyl alcohol | *v*_18_ + *v*_21_ – *v*_19_ = 0 |

*Variables in red indicate “overflow” fluxes (*cf.* red arrows in Fig. 1 of the *Main Text*).

Constraints on lignin composition along with numerical values are presented in Table S3. It is straightforward to translate them into a set of equality constraints in the form of Eq. 3 in the *Main Text*. To implement MOMA, we further define *δ*_i_ (see definition in the *Main Text*) in the following way: find the flux *v*_i_ whose catalyzing enzyme is modified, identify the percentage of the residual enzyme activity related to its wild-type level, and set *δ*_i_ to this number; unaffected fluxes have *δ*_i_ = 1.

We used *linprog* and *quadprog* routines in MATLAB to solve the linear and quadratic programming problems in FBA and MOMA, respectively.

**Table S3: Lignin content and monomer composition in wild-type and transgenic plants (see also Table S1 of ).**

*Percentages within the parentheses are the residual enzyme activity related to the wild-type level.

^†^T = H+G+S

**Identification of equivalent pathways**

Given the constraints in Eqs. 1-3 of the *Main Text*, we first perform an FBA for wild-type plants and then use this FBA-optimum as a reference in MOMA to infer the flux distribution for transgenic plants. A key issue that may arise from this approach is the existence of alternate optimal FBA solutions that give the same objective function value but with different flux distributions . To address this issue, we define an (16+2+1) × 24 matrix **A** and a (16+2+1)-dimensional vector **b** such that

(S1)

$$\mathbf{Av}=\mathbf{b}$$

collectively represents Eqs. 1 and 3 in the *Main Text*, as well as the normalization constraint *v*_1_ = 1. By this definition, we know that **v***^wt^* is a solution for the following problem:

$$\begin{matrix} z*=\mathbf{c}^{T}\mathbf{v} \\ \mathbf{Av}=\mathbf{b} \\ \mathbf{l}\leq\mathbf{v}\leq\mathbf{u} \end{matrix}$$

(S2)

where $z*=\mathbf{c}^{T}\mathbf{v}^{wt}$ is the optimal objective function value, and $\mathbf{l}$ and $\mathbf{u}$ are vectors of the lower and upper bounds on individual fluxes, respectively.

Apparently, alternate optima occur if there are solutions for Eq. S2 other than **v***^wt^*. If this is the case, the difference between an alternate solution and **v***^wt^*, defined as **w**, must also be a solution for the following sub-problem:

$$\begin{matrix} \mathbf{c}^{T}\mathbf{w}=0 \\ \mathbf{Aw}=\mathbf{0} \\ \mathbf{l}\leq\mathbf{v}^{wt}+\mathbf{w}\leq\mathbf{u} \end{matrix}$$

(S3)

because

(S4)

$$\mathbf{c}^{T}\mathbf{w}=\mathbf{c}^{T}(\mathbf{v}^{wt}+\mathbf{w})-\mathbf{c}^{\text{T}}\mathbf{v}=z*-z*=0$$

and

(S5)

$\mathbf{Aw}=\mathbf{A}(\mathbf{v}^{wt}+\mathbf{w})-\mathbf{Av}^{wt}=\mathbf{b}-\mathbf{b}=\mathbf{0}$.

If we define an (16+4) × 24 matrix $\mathbf{B}=\left[\begin{matrix} \mathbf{c}^{T} \\ \mathbf{A} \end{matrix}\right]$, then it is clear that $\mathbf{w}$ lies in the null space of $\mathbf{B}$, *i.e.*, $\mathbf{Bw}=\mathbf{0}$. Identification of the equivalent pathways, in this respect, is thus related to finding a meaningful basis of the null space of $\mathbf{B}$. By applying the Gauss-Jordan elimination to $\mathbf{B}$, we identified a basis for the pathway shown in Figure 1 of the *Main Text*; the vectors that constitute the basis are listed in Table S4 and also illustrated in Figure S6.

**Table S4: Basis Vectors (*BV*) for the Pathway Shown in Fig. 1 of the *Main Text***

|  | ***BV*1** | ***BV*2** | ***BV*3** | ***BV*4** |
| --- | --- | --- | --- | --- |
| ***v*_1_** | 0 | 0 | 0 | 0 |
| ***v*_2_** | 0 | 0 | 1 | 1 |
| ***v*_3_** | 0 | 0 | 1 | 1 |
| ***v*_4_** | 0 | 0 | 0 | 0 |
| ***v*_5_** | 0 | 0 | 0 | 0 |
| ***v*_6_** | 0 | 0 | 0 | 0 |
| ***v*_7_** | 0 | 0 | 0 | 1 |
| ***v*_8_** | 0 | 0 | 0 | 1 |
| ***v*_9_** | 0 | 0 | 0 | 1 |
| ***v*_10_** | -1 | 0 | 0 | 1 |
| ***v*_11_** | 1 | 0 | 0 | 0 |
| ***v*_12_** | -1 | 0 | 0 | 1 |
| ***v*_13_** | 1 | 0 | 0 | 0 |
| ***v*_14_** | 0 | 1 | 0 | 1 |
| ***v*_15_** | 0 | 0 | 0 | 0 |
| ***v*_16_** | 0 | -1 | 0 | 0 |
| ***v*_17_** | 0 | -1 | 0 | 0 |
| ***v*_18_** | 0 | -1 | 0 | 0 |
| ***v*_19_** | 0 | 0 | 0 | 0 |
| ***v*_20_** | 0 | 1 | 0 | 1 |
| ***v*_21_** | 0 | 1 | 0 | 0 |
| ***v*_22_** | 0 | 0 | -1 | -1 |
| ***v*_23_** | 0 | 0 | 1 | 0 |
| ***v*_24_** | 0 | 0 | 0 | 1 |


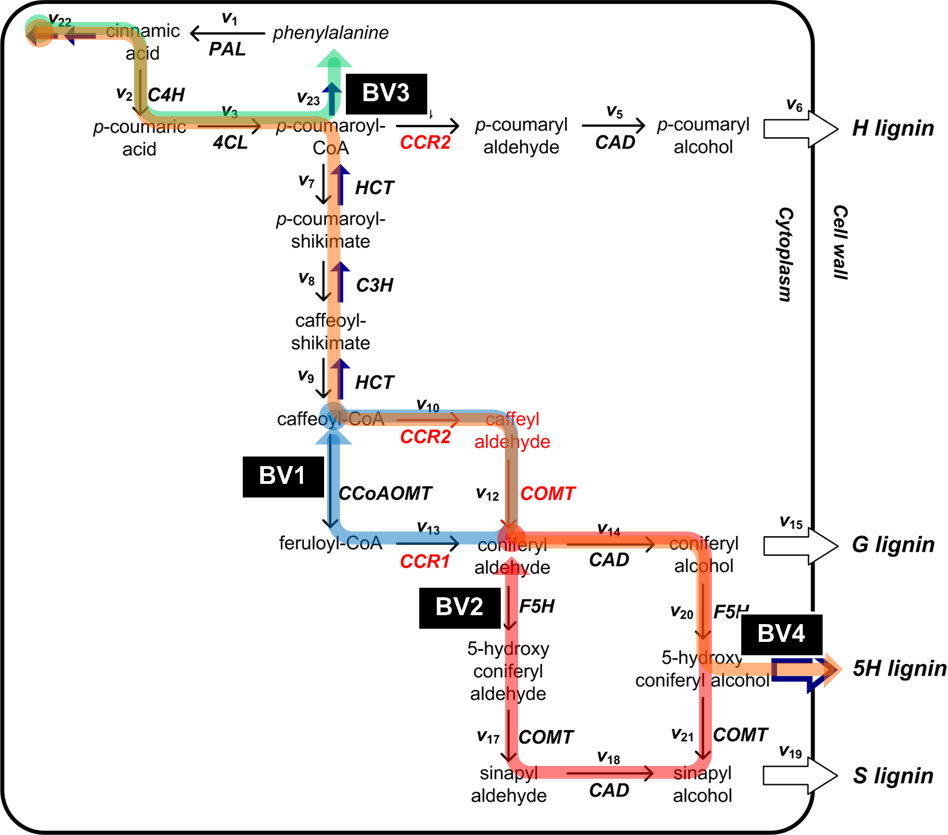


**Figure S6. Illustration of the four basis vectors.**

Two observations are made from the identified basis. First, *BV*1 and *BV*2 correspond to the two inner loops within the pathway. Second, both *BV*3 and *BV*4 have non-zero components corresponding to two overflow fluxes, with one being positive and the other one negative. Since the three overflow fluxes are presumably minimized in wild-type plants and thus set to a small positive number in the original FBA-derived optimum **v***^wt^*, any perturbation **w** involving a non-trivial linear combination of *BV*3 and *BV*4 cannot be a solution for the system S3 because adding a negative value to one of these overflow fluxes would make it smaller than the lower bound. Thus, a valid perturbation **w** can be represented as:

$$\begin{matrix} \mathbf{w}=\alpha_{1}BV1+\alpha_{2}BV2\text{ subject to} \\ \mathbf{1}\leq\mathbf{v}^{wt}+\mathbf{w}\leq\mathbf{u}\text{ and }\alpha_{1},\alpha_{2}\in\mathbf{R} \end{matrix}$$

(S6)

The two sets of equivalent pathways as specified by *BV*1 and *BV*2 are (*v*_10_ →*v*_12_, *v*_11_→*v*_13_) and (*v*_14_→*v*_20_→*v*_21_, *v*_16_→*v*_17_→*v*_18_). To identify a unique, physiologically relevant flux distribution for wild-type plants, we used the maximum activities of two *Medicago* CCR isoforms (Table S5) to constrain the first two equivalent pathways with the following constraint: $v_{10}/v_{13}=0.35/1.64$. The constraint is justified because, assuming that the two CCR-catalyzed reactions are described by Michaelis-Menten kinetics and that the levels of both CoA esters are well below the corresponding Michaelis constraints (54.5 μM for feruloyl CoA and 23.4 μM for caffeoyl CoA; ), the ratio between the two CoA esters is approximately

$\frac{[\text{Caffeoyl CoA}]}{[\text{Feruloyl CoA}]}\cong\frac{v_{10}}{v_{13}}\times\frac{1.64}{0.35}\times\frac{23.4}{54.5}\cong0.43$,

(S7)

which is consistent with the prediction in potato tubers that feruloyl CoA is more abundant than caffeoyl CoA .

Since all the enzymes implicated in the other two equivalent pathways have yet been characterized for *Medicago*, we instead used the maximum activities of *Arabidopsis* F5H to set up the constraint: $v_{16}/v_{20}=5/6$. Notice that this approximation is not an important issue because all the main results and postulates still hold whether or not the later constraint is applied (data not shown).

**Table S5: Documented Enzyme Kinetic C**onstants for CCR and F5H.

| **Enzyme** | **Gene** | **Substrate** | ***V*_max_** | **Reference** |
| --- | --- | --- | --- | --- |
| Cinnamoyl-CoA reductase (CCR) | MtCCR1 | Feruloyl CoA | 1.64^a^ |  |
|  | MtCCR2 | Caffeoyl CoA | 0.35^a^ |  |
| Ferulate 5-hydroxylase (F5H) | FAH1^c^ | Coniferyl aldehyde | 5^b^ |  |
|  |  | Coniferyl alcohol | 6^b^ |  |

^a^Unit in μmol/min

^b^Unit in pkat/mg; kat = mol/s

^c^The gene encoding ferulate 5-hydroxulase was cloned in *Arabidopsis*

Interestingly, the three major monolignols (H, G, and S) are not involved in the basis vectors. A possible reason is the following: The three fluxes *v*_6_, *v*_15_, and *v*_19_ are more or less fixed by the normalization (*v*_1_ = 1) and the two “proportion” constraints in Eq. 3 of the *Main Text*, if the task is to maximize their sum (or equivalently, to minimize the sum of three “overflow” fluxes). As a result, their values would not be influenced by the different weighting of equivalent pathways, whereas values of some other intermediate fluxes would.

**II. Kinetic analysis of a reduced model**

In order to validate the results from the flux-based analysis in some independent fashion, we generated an ensemble of ordinary differential equation (ODE) models for the core of the pathway (Figure S7) that controls the relative proportion of G and S lignin. Using a standard formulation with simplified variable names and Michaelis-Menten functions for each enzymatic step, we defined

$$\begin{matrix} \frac{dX_{1}}{dt}=\frac{V_{1}I}{I+K_{1}}-\frac{V_{3}X_{1}}{X_{1}+K_{3}} \\ \frac{dX_{2}}{dt}=\frac{V_{2}I}{I+K_{2}}-\frac{V_{4}X_{2}}{X_{2}+K_{4}} \\ \frac{dX_{3}}{dt}=\frac{V_{3}X_{1}}{X_{1}+K_{3}}+\frac{V_{4}X_{2}}{X_{2}+K_{4}}-\frac{V_{5}X_{3}}{X_{3}+K_{5}}-\frac{V_{7}X_{3}}{X_{3}+K_{7}} \\ \frac{dX_{4}}{dt}=\frac{V_{5}X_{3}}{X_{3}+K_{5}}-\frac{V_{6}X_{4}}{X_{4}+K_{6}}-\frac{V_{8}X_{4}}{X_{4}+K_{8}} \end{matrix}$$

(S8)

where *K_i_*’s are Michaelis constants and *V_i_*’s are maximum rates. To ensure that the search was representative of the parameter space, we sampled 10,000 sets of kinetic parameters uniformly over logarithmic scales, using the Latin hypercube sampling method. The sampling ranges were *V_i_* ~0.1-10 and *K_i_* ~0.1-10. Furthermore, in order to account for the possibility of cooperative binding, we replaced $V_{i}S/(S+K_{i})$ in Eq. S8 with Hill functions of the type $V_{i}S^{n}/(S^{n}+K_{i}^{n})$ and sampled the Hill coefficient *n* from the range 1-4.


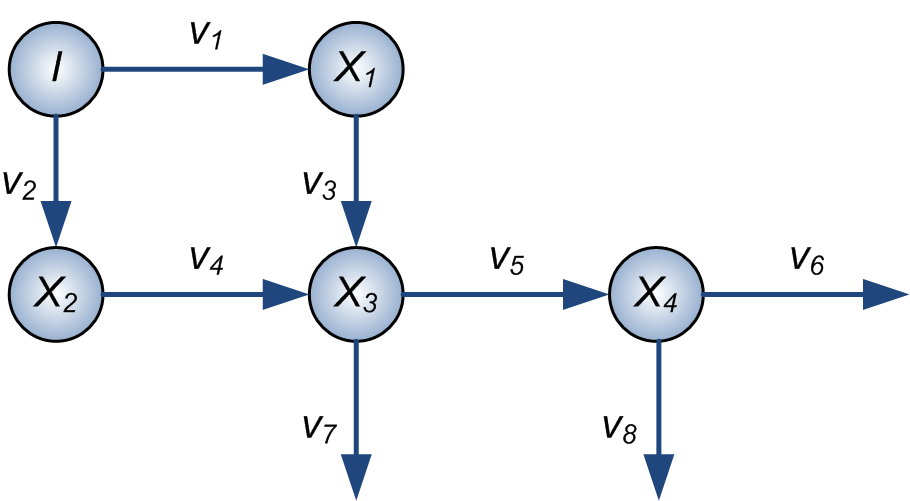


**Figure S7. Simplified network with one fixed input (*I*) and four metabolites (*X_1_*-*X_4_*), which was used as a reduced model for studying the roles of CCR1 and CCR2 in the monolignol pathway. Metabolic fluxes, denoted as *v_1_*-*v_8_*, are represented by arrows that connect metabolites or leave the system. Each kinetic parameter in Eq. S13 is numbered by the corresponding flux. Reactions $v_{1},\ldots,v_{5}$ correspond to CCR2, CCoAOMT, COMT, CCR1, and CAD, respectively. *v_6_* represents transport into the cell wall, and *v_7_* and *v_8_* represent F5H. Pools *I*, *X*_1_, …, *X*_4_ correspond to caffeoyl-CoA, caffeoyl aldehyde, feruloyl-CoA, coniferyl aldehyde, and conferyl alcohol, respectively.**

Each sampled parameter set defines a kinetic model with which we can simulate different cases of genetic modifications and monitor how the S/G ratio responds. First, we numerically determined a steady state by solving the ODEs with all dependent variables in the network, as well as the input *I*, set to a concentration of 1. Gene modifications were modeled by decreasing the *V_i_* of the targeted enzyme (*e.g.*, *V*_2_ for CCoAOMT). With this adjustment, we solved the ODEs again and then computed the S/G ratio as

$\text{S}/\text{G}ratio\approx\frac{\bar{v}_{7}+\bar{v}_{8}}{\bar{v}_{6}}=\frac{\frac{V_{7}\bar{X}_{3}}{\bar{X}_{3}+K_{7}}+\frac{V_{8}\bar{X}_{4}}{\bar{X}_{4}+K_{8}}}{\frac{V_{6}\bar{X}_{4}}{\bar{X}_{4}+K_{6}}}$,

(S9)

where variables with bars indicate steady-state values. The further analysis excluded ill-behaved models, which were defined as systems spending an unduly large amount of time approaching the post-modification steady state, or systems in which one or more metabolites were depleted during the transition. The remaining admissible models were evaluated for their ability to change the S/G ratio; an increase in the S/G ratio was deemed significant if it was greater than 50%.

**References**

1. Chen F, Reddy MSS, Temple S, Jackson L, Shadle G, et al. (2006) Multi-site genetic modulation of monolignol biosynthesis suggests new routes for formation of syringyl lignin and wall-bound ferulic acid in alfalfa (*Medicago sativa* L.). Plant J 48: 113-124.

2. Lee S, Phalakornkule C, Domach MM, Grossmann IE (2000) Recursive MILP model for finding all the alternate optima in LP models for metabolic networks. Comput Chem Eng 24: 711-716.

3. Mahadevan R, Schilling CH (2003) The effects of alternate optimal solutions in constraint-based genome-scale metabolic models. Metab Eng 5: 264-276.

4. Zhou R, Nakashima J, Jackson L, Shadle G, Temple S, et al. (2010) Distinct cinnamoyl CoA reductases involved in parallel routes to lignin in *Medicago truncatula*. Proc Natl Acad Sci U S A 107: 17803-17808.

5. Heinzle E, Matsuda F, Miyagawa H, Wakasa K, Nishioka T (2007) Estimation of metabolic fluxes, expression levels and metabolite dynamics of a secondary metabolic pathway in potato using label pulse-feeding experiments combined with kinetic network modelling and simulation. Plant J 50: 176-187.

6. Humphreys JM, Hemm MR, Chapple C (1999) New routes for lignin biosynthesis defined by biochemical characterization of recombinant ferulate 5-hydroxylase, a multifunctional cytochrome P450-dependent monooxygenase. Proc Natl Acad Sci U S A 96: 10045-10050.
